# Research Review: The impact of the COVID‐19 pandemic on the mental health of children and young people with pre‐existing mental health and neurodevelopmental conditions – a systematic review and meta‐analysis of longitudinal studies

**DOI:** 10.1111/jcpp.14117

**Published:** 2025-01-30

**Authors:** Brian C. F. Ching, Johnny Downs, Shuo Zhang, Hannah Abdul Cader, Jessica Penhallow, Elvina Voraite, Teodora Popnikolova, Alice Wickersham, Valeria Parlatini, Emily Simonoff

**Affiliations:** ^1^ Department of Child and Adolescent Psychiatry, Institute of Psychiatry, Psychology and Neuroscience King's College London London UK; ^2^ National Institute for Health Research (NIHR) Biomedical Research Centre South London and Maudsley NHS Foundation Trust London UK

**Keywords:** COVID‐19, pandemic, children and young people, longitudinal, mental health, neurodevelopmental

## Abstract

**Background:**

Systematic reviews have suggested mixed effects of the COVID‐19 pandemic on the mental health of children and young people. However, most included studies focused on the general population and were cross‐sectional. The long‐term impact on those with pre‐existing mental health and/or neurodevelopmental conditions remains unclear. Thus, we conducted a systematic review and meta‐analysis to examine the longitudinal impact of the pandemic on the mental health of this clinical population and potential explanatory factors.

**Methods:**

Ovid Medline, Embase, APA PsycInfo and Global Health databases were searched between 1 January 2020 and 3 August 2023 (PROSPERO CRD42022383546). We included longitudinal studies that compared mental health symptoms between pre‐ and during pandemic and/or during pandemic timepoints in children and young people (≤18 years old) with pre‐existing mental and/or neurodevelopmental conditions. Outcomes included internalising, externalising and other symptoms. Risk of bias was rated using an adapted tool. Included studies were narratively synthesised and multi‐level meta‐analyses were conducted where the number of studies was sufficient.

**Results:**

We identified 21 studies (*N* = 2,617) from 6,083 records. Studies differed across countries, diagnoses, measures, informants and timepoints. All had overall moderate‐to‐high risk of bias. Narrative synthesis found mixed evidence of symptom change, with individual studies showing increase/reduction/no change. Factors such as diagnosis, baseline symptom severity, age and sex/gender may explain variation in outcomes. Multi‐level meta‐analyses were feasible for a limited number of outcomes and found no significant changes in internalising and externalising symptoms pre‐ versus during pandemic or internalising symptoms between 2020 pandemic phases, and high heterogeneity was noted.

**Conclusions:**

The impact of the pandemic on the mental health of children and young people with pre‐existing conditions varied according to individual and contextual vulnerabilities, which were not fully captured in pooled analyses. Further research needs to investigate longer‐term impacts and better stratify this vulnerable population.

## Introduction

The COVID‐19 pandemic with its disruptions, including school closures and social distancing measures, has uniquely hindered opportunities for children and young people to achieve fundamental cognitive, emotional and social milestones critical for healthy development (Garagiola et al., 2022). These milestones include starting school, forming social identities, navigating transition from primary and secondary school and taking state examinations. The scale of these disruptions was unprecedented and may have introduced biopsychosocial risks for long‐term developmental and mental health outcomes (Benner & Mistry, 2020; Sonuga‐Barke, 2021).

Systematic reviews of the general population found mixed evidence of mental health impacts: individual studies reported symptom increases and decreases (Ahmed et al., 2023; Newlove‐Delgado et al., 2023), whereas pooled estimates from meta‐analyses were largely inconclusive, providing no clear evidence that pandemic disruptions have had overall positive or negative impacts on children and young people's mental health (Newlove‐Delgado et al., 2023; Robinson, Sutin, Daly, & Jones, 2022; Sun et al., 2023). Cross‐sectional studies suggest that children and young people with pre‐existing mental health and neurodevelopmental conditions may have experienced high levels of internalising and externalising symptoms during the pandemic (e.g. Parlatini et al., 2023; Waite et al., 2021), possibly more than youth without pre‐existing conditions (e.g. Waite et al., 2021). Mental health service data showed an overall increase in demands during the pandemic (McNicholas et al., 2021; Wong et al., 2023), further highlighting increased mental health needs among children and young people.

Children and young people with pre‐existing conditions appear differentially impacted over the course of the pandemic, possibly faring less well than those without established mental health conditions (Pierce et al., 2021). Those with pre‐existing conditions may carry a higher burden of risk factors or be more sensitive to exacerbation by pandemic restrictions, for example increasing their exposure to discordant family relations, limiting access to outside space and depriving access to additional educational support (Parlatini et al., 2023). Pandemic restrictions may also be additive, further impairing children with pre‐existing conditions to make functional adaptations to meet pre‐existing needs, resulting in worsening mental health outcomes.

Whilst most pandemic research focused on children and young people in the general population, it is important to investigate whether effects are similar for those with pre‐existing conditions. Focusing on clinical samples provides an opportunity to compare effects across conditions and other clinical characteristics, which are usually not possible in general samples. Population‐cohort studies have demonstrated that there may be significant differences in mental health outcomes during pandemic by sociodemographic characteristics (e.g. Kwong et al., 2021; Pierce et al., 2021; Ravens‐Sieberer et al., 2022), but less is known of their role in clinical populations. Parlatini et al. (2023) found significant differences in emotional and behavioural outcomes across diagnostic groups in the first lockdown in the UK, which suggests complex variability in the way the pandemic was experienced by children with pre‐existing conditions. Investigating mental health changes and explanatory factors in clinical populations during the pandemic can inform policy planning, service provision and clinical workforce preparedness.

Moreover, the pandemic's longitudinal impact on the mental health of children and young people with pre‐existing conditions remains unclear. Firstly, reviews in children and young people mostly included cross‐sectional studies or assessed longitudinal mental health changes only up until late 2020. Secondly, potential explanatory factors of mental health changes during the pandemic have not been comprehensively investigated. To our knowledge, this is the first systematic review and meta‐analysis to investigate the longitudinal impact of the pandemic on the mental health of children and young people with pre‐existing mental health and neurodevelopmental conditions.

## Methods

This systematic review was preregistered on PROSPERO (CRD42022383546) and followed the Preferred Reporting Items for Systematic Reviews and Meta‐Analyses (PRISMA) guidelines (Page et al., 2021).

### Eligibility criteria

Longitudinal studies reporting mental health outcomes in children and young people (≤18 years old at follow‐up) with pre‐existing mental health and/or neurodevelopmental conditions were included. Studies were deemed eligible if children and young people were clinically diagnosed according to the Diagnostic and Statistical Manual of Mental Disorders (American Psychiatric Association, 2013) or International Classification of Diseases (World Health Organization, 2019); if they scored above clinical threshold on validated measures; or if they attended mental health services pre‐pandemic. Studies reporting data on mixed samples were eligible if at least 50% of the sample were ≤18 and child data could be extracted. We included studies that compared outcomes pre‐ versus during pandemic, and/or between during pandemic timepoints, to examine how mental health changed during the pandemic compared to pre‐pandemic and the potentially variable and temporal changes during the pandemic. See Appendix S1 for further information on the eligibility criteria and Appendix S2 for how outcomes were aggregated. Primary or secondary quantitative research from any country was eligible if peer‐reviewed and in English and conference abstracts if sufficient data available. Authors of eligible abstracts were contacted about published studies.

### Search strategy

Ovid Medline, Embase, APA PsycInfo and Global Health databases were searched between 1 January 2020 and 3 August 2023 (see Appendix S3 for the search strategy). Identification of studies via citation searching and Google Scholar were also used.

### Selection process

After removing duplicates, titles and abstracts and potentially eligible full‐text papers were double screened independently (BCFC, SZ, HAC, JP, EV and TP). Substantial agreements were found between raters at title/abstract (*kappa* = 0.92) and full‐text (*kappa* = 0.65) phases. Discrepancies were discussed and resolved with a third independent reviewer (VP).

### Data extraction

Data extracted included study and sample characteristics, mental health outcomes, assessment timepoints and summary of findings. See Appendix S4 for further information on data extraction.

### Restriction levels

National pandemic data was assessed using the Oxford COVID‐19 Government Response Tracker (OxCGRT; Hale et al., 2021), which quantified the stringency of country‐specific government mandated restrictions across time (see Appendix S4).

### Risk of bias

Like other published reviews (e.g. Robinson et al., 2022), previous study quality appraisal and risk of bias assessment scales were reviewed, and a list of risk‐of‐bias indicators were extracted and adapted. BCFC and SZ independently conducted the critical appraisal using selected indicators, resolved discrepancies in discussion and provided an overall quality rating (see Appendix S5 for detailed information on risk of bias indicators). Potential publication bias was inspected using funnel plots and Egger's regression test.

### Synthesis methods

We conducted narrative synthesis on all included studies and relevant outcomes. Findings comparing mental health outcomes (a) pre‐ versus during pandemic and (b) during pandemic phases were summarised by symptom categories. To allow meaningful comparisons, the pandemic timeline captured by each study was categorised into three ‘phases’: acute (March 2020–June 2020), remission (July 2020–December 2020) and resurgence (January 2021–June 2021). Only one study presented data beyond June 2021 (Prato et al., 2023). If studies had data in multiple timepoints within each phase, the data from the month where most other studies had data from was selected. We reported the statistical findings of individual studies; we refer to change (and indicate the direction) when significant, and no change if non‐significant. Explanatory risk factors were synthesised where possible.

Meta‐analyses were conducted in R 4.3.1 (R Core Team, 2023) where at least 5 comparable studies were available. We considered studies comparing continuous mental health outcomes (a) pre‐ versus during pandemic and (b) during pandemic separately. We conducted multi‐level meta‐analyses to account for correlated effects in studies reporting multiple outcomes, which allows for nested effects within levels (i.e. individual participants, individual studies and parent and young people reported data and internalising/externalising symptom outcomes within each study), consistent with previous pandemic‐related reviews (e.g. Robinson et al., 2022). We opted for random‐effects models to enhance the generalisation of findings (Cheung, Ho, Lim, & Mak, 2012). We calculated standardised mean change (SMC) for each comparison of interest to assess the amount of change within‐sample (Viechtbauer, 2010). Percentage of heterogeneity due to sampling variance (level 1), within‐study (level 2), and between‐study (level 3) variability were assessed using the multi‐level *I* ^2^ statistic. Subgroup analyses on symptom type, diagnostic group, informant and age were conducted where appropriate. Sensitivity analyses including combined symptom measures were conducted to assess robustness. Forest plots were used to visualise individual and pooled SMCs and 95% confidence intervals (CIs). See Appendix S6 for further statistical information.

## Results

### Summary of included studies

**Figure 1:**
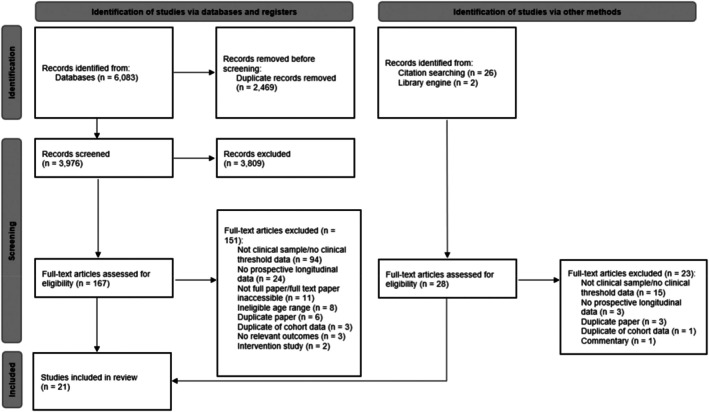
PRISMA flow diagram

The search was first conducted on 11 December 2022 and updated on 3 August 2023. These identified 6,083 records, and 21 studies (*N* = 2,617) met inclusion criteria (see Figure 1 for PRISMA flow diagram). Studies were conducted in the United States (seven), Italy (five), United Kingdom (three), Australia (two), Netherlands (two), Japan (one) and Spain (one). Sample sizes (*N* = 11–780), diagnostic group (ADHD, autism spectrum disorder (ASD), mixed neurodevelopmental conditions, neuropsychiatric conditions and others), follow‐up duration (range from ~1 to 64 months), and outcomes varied considerably.

Twelve studies assessed mental health outcomes pre‐ and during pandemic and 14 studies assessed mental health outcomes at two or more pandemic timepoints. Eighteen studies assessed mental health outcomes in the acute (March–April 2020), 15 studies in the remission (July–December 2020), and seven studies in the resurgence phase (January–June 2021). Pandemic restrictions across timepoints were relatively stable (most ranged from 60–80, moderate‐to‐high restrictions). See Table 1 for more details of characteristics of included studies, Table 2 for summary of key findings, Figure S1 for timeline of included studies, Appendix S7 for further information on how studies were treated for the meta‐analyses, and Appendix S8 for detailed information on narrative syntheses findings.

**Table 1:** Summary of included study characteristics

Author (year)	Country	Sample and setting	Baseline, *N*	Diagnostic criteria	Baseline mean age (*SD*), range	Gender	Ethnicity	Pre‐COVID timepoint	Follow‐up timepoint(s)	Follow‐up duration	Attrition at follow‐up	COVID restrictions at timepoint(s)	Relevant outcome(s) (measure(s))	Informant(s)
Bouter et al. (2023)	Netherlands	High‐risk adolescents form iBerry cohort study Schools	61	Clinical threshold in self‐report measures	15.10 (0.94)	24 boys, 37 girls	43 Dutch, 12 non‐Dutch	September 2015–September 2019	April 2020 January 2021	~64 months	N/A	65 65	Depressive symptoms, anxiety symptoms, oppositional defiant symptoms, psychotic symptoms and suicidality (YSR)	YP
Breaux et al. (2021)	United States	Adolescents with ADHD from a prospective longitudinal study Schools	118	Clinical diagnostic assessment	15–17^a^	132 boys, 106 girls^a^	82% White, 7% mixed, 6% Black, 4% Asian, 1% other; 4% Hispanic/Latinx^a^	September 2018–February 2020	May–June 2020 July–August 2020	~5–23 months	N/A	65 65	Depressive symptoms, anxiety symptoms (RCADS) and inattention, hyperactivity/impulsivity and oppositionality/defiance (VADRS)	YP Parent
Conti et al. (2020)	Italy	Children and adolescents with neuropsychiatric disorders Mental health services	141 (61 1.5–5 years old, 80 6–18 years old)	Clinical diagnostic assessment	1.5–18	117 boys, 24 girls	N/A	September 2019–February 2020	April–May 2020	~2–8 months	N/A	80	Internalising symptoms, externalising symptoms, depressive symptoms, anxiety symptoms, inattention/hyperactivity and conduct problems (CBCL 1.5–5 and CBCL 6–18)	Parent
Corbett et al. (2021)	United States	Autistic adolescents Schools, mental health services and the community	61	Clinical diagnostic assessment	13.23	46 boys, 15 girls	84.42% White, 4.92% African American, 10.66% mixed^a^	April–May 2019	April–May 2020 August–September 2020	~15 months	N/A	65 65	Anxiety symptoms (State–Trait Anxiety Inventory for Children, STAI‐C)	YP
Day et al. (2023)	United States	Autistic children and adolescents University research database	51	Clinical diagnostic assessment	12.9 (2.57), 8.50–17.4	41 boys, 10 girls	43 White, 4 Mixed, 2 Black, 1 Asian, 1 Hispanic/Latinx	N/A	June–September 2020 every 2 weeks until December 2020	~3–5 months	N/A	65 65	Internalising symptoms (RCADS)	YP Parent
De Giacomo et al. (2021)	Italy	Children with neurodevelopmental disorders Mental health services	71 (14 ASD, 7 ADHD, 22 other neurodevelopmental disorders)	Clinical diagnostic assessment	9 (3.67)	52 boys, 19 girls	N/A	Pre‐COVID (1 year before pandemic)	April–July 2020	~16 months	N/A	70	Internalising and externalising symptoms (CBCL)	Parent
de Maat et al. (2023)	Netherlands	Autistic children and adolescents from the ASD & COVID‐19 study Mental health services	62	Clinical diagnostic assessment	12.8 (4.5)	74.2% boys, 25.8% girls	75.6% Dutch, 24.4% other	March 2019–March 2020	January–May 2021	~10–26 months	*N* = 7 were removed from analysis because of incomplete data across timepoints.	65	Emotional and behavioural problems (BPM)	Parent
Dvorsky et al. (2022)	United States	Adolescents with ADHD Schools	118	Clinical diagnostic assessment	15–17^a^	132 boys, 106 girls^a^	82% White, 7% mixed, 6% Black, 4% Asian, 4% Latinx, 1% other^a^	N/A	May–June 2020 July–August 2020 October–November 2020	~6 months	N/A	65 65 65	Pandemic‐related depressive and anxiety symptoms (CRISIS)	YP
Fredrick et al. (2022)	United States	Adolescents with ADHD from a prospective longitudinal study Schools	118	Clinical diagnostic assessment	15–17^a^	132 boys, 106 girls^a^	82% White, 7% mixed, 6% Black, 4% Asian, 1% other; 4% Hispanic/Latinx^a^	N/A	May–June 2020 March–April 2021	~9–11 months	N/A	65 60	Anxiety symptoms, depressive symptoms (RCADS)	YP Parent
Hall, Marston, et al. (2023)	United Kingdom	Children and adolescents with tic disorder Mental health services, charity and self‐referrals	47	Clinical diagnostic assessment	12.5 (2.3)	33 boys, 14 girls	42 White	March–October 2019	March–October 2020	~12 months	Complete case analysis	65	Tic severity and impairment (YGTSS)	Clinician
Hall, Partlett, et al. (2023)	United Kingdom	Children and adolescents with ADHD and/or ASD from the Co‐SPACE study Social media, charities and partner organisations	780 (160 ADHD, 465 ASD, 155 ADHD and ASD)	Self‐reported diagnosis	10.3–10.6 (3.1–3.4)	530 boys, 234 girls, 16 other/prefer not to say	714 White, 43 Mixed, 6 Asian, 3 Black, 14 other/prefer not to say	N/A	March–April 2020 April–May 2020	~1–2 months	0%	60 60	Emotional symptoms, conduct problems, inattention/hyperactivity (SDQ) and pandemic‐related anxiety (PAS)	Parent
Houghton et al. (2022)	Australia	Children and adolescents with neurodevelopmental disorders from a larger longitudinal study Schools	238 (76 ADHD, 36 ASD, 134 SLD, 19 other)	Clinical diagnostic assessment	13.52 (1.44), 10–16	131 boys, 107 girls	N/A	November 2018–April 2019	March 2020 July/August 2020	~20 months	54% at March 2020	40 70	Depression symptoms (CDI:SR), internalising symptoms and externalising symptoms (SDQ)	YP
Lewis et al. (2023)	United States	Adolescents with ADHD or internalising disorders from a prospective longitudinal study Schools	236 (117 ADHD, 38 depression or anxiety)	Clinical diagnostic assessment	16.74 (0.60), 15–17	130 boys, 106 girls	190 White, 23 mixed, 13 Black, 10 Asian	N/A	May–June 2020 July–August 2020 October–November 2020 March–May 2021	~8–11 months	22% missing data at last timepoint	65 65 65 60	Post‐traumatic stress symptoms (CATS)	YP
Lugo‐Marín et al. (2021)	Spain	Autistic children and adolescents Mental health services	37	Clinical diagnostic assessment	10.7 (3.4)	32 boys, 5 girls	N/A	Pre‐COVID	May–July 2020	~2 months	1 did not complete CBCL	60	Internalising symptoms, externalising problems, depressive symptoms, anxiety symptoms, inattention/hyperactivity, oppositional defiant problems (CBCL)	Parent
Prato et al. (2023)	Italy	Children and adolescents with tic disorder Mental health services	11	Clinical diagnostic assessment	14.8 (2.6)	27.3% boys, 72.7% girls	N/A	N/A	June 2021–June 2022 (6 and 12 months after enrolment)	12 months	0%	65 65	Depressive symptoms (CDI), anxiety symptoms (MASC), tic severity and impairment (YGTSS) and OCD symptoms (CY‐BOCS)	YP Clinician
Raffagnato et al. (2021)	Italy	Children and adolescents with affective, behavioural and neurodevelopmental disorders Mental health services	39	Clinical diagnostic assessment	13.3 (2.84), 11–18	14 boys, 25 girls	N/A	N/A	April–May 2020 August–September 2020	~4 months	70% (17 dropped out)	80 65	Internalising symptoms, externalising symptoms and post‐traumatic stress symptoms (CBCL and YSR)	YP Parent
Sadeghi et al. (2022)	United States	Adolescents with past or current depression from the NIMH CAT‐D cohort study Self‐referral and practitioner referral	96/97 (completed assessments for depressive and anxiety symptoms, respectively)	Clinical diagnostic assessment	15.68	28 boys, 68 girls	64 White, 8 Black, 12 Mixed, 10 Asian, 1 Native, 1 declined to answer	March 2019–March 2020	March–April 2020 April–May 2020 May 2020 May–June 2020 June–July 2020 July–September 2020 September 2020–January 2021 January–March 2021	~24 months	0%	60 65 65 65 65 65 65 65	Depressive symptoms (MFQ) and anxiety symptoms (SCARED and SCARED‐short)	YP Parent
Siracusano et al. (2021)	Italy	Autistic children and adolescents Mental health services	85 (33 2–5 years old, 52 6–18 years old)	Clinical diagnostic assessment	7, 2–18	68 boys, 17 girls	N/A	Pre‐COVID	May–July 2020	~9 months	N/A	65	Internalising symptoms and externalising symptoms (CBCL)	Parent
Summerton et al. (2023)	Australia	Children and adolescents with ADHD from the ADHD COVID‐19 study Social media, partner organisations and ADHD parent support groups	207	Self‐reported diagnosis	10.6 (3.0), 6–17	106 boys, 34 girls	N/A	N/A	May 2020 May–June 2021	~12–14 months	70% completed follow‐up	60 55	Depressive symptoms (SMFQ), anxiety symptoms (SCAS), irritability (ARI), inattention, hyperactivity/impulsivity and oppositional defiant symptoms (SNAP‐IV)	Parent
Suzuki and Hiratani (2021)	Japan	Children and adolescents with a neurodevelopmental disorder Mental health services	143	N/A	9.76 (2.36), 6–16	118 boys, 25 girls	N/A	April 2018–February 2020	October–December 2020	~9–29 months	N/A	45	Irritability and hyperactivity (ABC‐J)	Parent
Toseeb and Asbury (2023)	United Kingdom	Autistic (and other SENs) children and adolescents Schools, partner organisations and self‐referral	250	Self‐reported diagnosis	11, 5–18	70% boys, 30% girls	90% White British	N/A	March–April 2020 April 2020–May 2020 May 2020–June 2020 September–October 2020	~6 months	63% at last timepoint (missing data was addressed using maximum likelihood estimator)	60 60 65 60	Depressive symptoms (RCADS) and anxiety symptoms (adapted RCADS)	Parent

Only data available in each included study are presented here.

^a^ Data is from the whole sample of the study, including comparators.

**Table 2:** Key findings from individual studies

Author (year)	Pre‐COVID timepoint	Follow‐up timepoint(s)	Baseline, *N*	Relevant outcome(s) (measure(s))	Informant(s)	Summary of findings
Bouter et al. (2023)	September 2015–September 2019	April 2020 January 2021	61	Depressive symptoms, anxiety symptoms, oppositional defiant problems, psychotic symptoms and suicidality (YSR)	YP	Depressive symptoms increased significantly between pre and April 2020 (acute) in high‐risk adolescents. 19.3% increased and 9.9% decreased. Anxiety symptoms decreased significantly between pre and April 2020 (acute) in high‐risk adolescents. 8.5% increased and 8.5% decreased. Oppositional defiant symptoms did not change between pre and April 2020 (acute) in high‐risk adolescents. 5.6% increased and 3.8% decreased. Psychotic symptoms decreased significantly between pre and April 2020 (acute) in high‐risk adolescents. Suicidality did not change between pre and April 2020 (acute) in high‐risk adolescents. Depressive symptoms increased between April 2020 (acute) and January 2021 (resurgence) in high‐risk adolescents. 20.5% increased and 7.3% decreased. Anxiety symptoms did not change between April 2020 (acute) and January 2021 (resurgence) in high‐risk adolescents. 10% increased and 4.4% decreased. Oppositional defiant symptoms did not change between April 2020 (acute) and January 2021 (resurgence) in high‐risk adolescents. 2.6% increased and 4.7% decreased. Psychotic symptoms decreased between April 2020 (acute) and January 2021 (resurgence) in high‐risk adolescents. Suicidality did not change between April 2020 (acute) and January 2021 (resurgence) in high‐risk adolescents.
Conti et al. (2020)	September 2019–February 2020	April–May 2020	141 (61 1.5–5 years old, 80 6–18 years old)	Internalising symptoms, externalising symptoms, depressive symptoms, anxiety symptoms, inattention/hyperactivity, oppositional defiant problems, obsessive–compulsive symptoms and post‐traumatic stress symptoms (CBCL 1.5–5 and CBCL 6–18)	Parent	Internalising and depressive symptoms did not change between pre and April–May 2020 (acute) in 1.5–5‐ or 6–18‐year‐old children and adolescents with neuropsychiatric disorders. Anxiety symptoms increased significantly between pre and April–May 2020 (acute) in 1.5‐5‐year‐olds but did not change in 6–18‐year‐old children and adolescents with neuropsychiatric disorders. Externalising symptoms, inattention/hyperactivity problems and conduct problems did not change between pre and April–May 2020 (acute) in old children and adolescents with neuropsychiatric disorders. Obsessive–compulsive problems increased significantly between pre and April–May 2020 (acute) in 6–18‐year‐old children and adolescents with neuropsychiatric disorders. Post‐traumatic stress problems increased significantly between pre and April–May 2020 (acute) in 6‐18‐year‐old children and adolescents with neuropsychiatric disorders.
Corbett et al. (2021)	April–May 2019	April–May 2020 August–September 2020	61	Anxiety symptoms (State–Trait Anxiety Inventory for Children, STAI‐C)	YP	Anxiety symptoms did not change between pre‐pandemic and April–May 2020 (acute) or August–September 2020 (remission) in autistic adolescents. Anxiety symptoms did not change between April–May 2020 (acute) and August–September 2020 (remission) in autistic adolescents.
Day et al. (2023)	N/A	June–September 2020 every 2 weeks until December 2020	51	Internalising symptoms (RCADS)	YP Parent	Internalising symptoms significantly decreased between June–September and December 2020 in autistic children and adolescents.
De Giacomo et al. (2021)	Pre‐COVID (1 year before pandemic)	April–July 2020	71 (14 ASD, 7 ADHD, 22 other neurodevelopmental disorders)	Internalising and externalising symptoms (CBCL)	Parent	Internalising symptoms did not significantly change between pre‐pandemic and April–July 2020 (acute/remission) in children with neurodevelopmental conditions. Externalising symptoms did not significantly change between pre‐pandemic and April–July 2020 (acute/remission) in children with neurodevelopmental conditions.
de Maat et al. (2023)	March 2019–March 2020	January–May 2021	62	Emotional and behavioural problems (BPM)	Parent	Emotional and behavioural problems did not significantly change between pre‐pandemic and January–May 2021 (resurgence) in autistic children and adolescents. Levene's test indicated that there were significantly higher interindividual variability in emotional and behavioural problems during the pandemic in the autistic group compared to children without. 32 children experienced an increase and 30 experienced no change or a decrease in emotional and behavioural problems.
Hall, Marston et al. (2023)	March–October 2019	March–October 2020	47	Tic severity and impairment (YGTSS)	Clinician	Tic severity and impairment did not significantly change between pre‐pandemic and March–October 2020 (acute/remission) in children and adolescents with tic disorder.
Hall, Partlett, et al. (2023)	N/A	March–April 2020 April–May 2020	780 (160 ADHD, 465 ASD, 155 ADHD and ASD)	Internalising symptoms, conduct problems, inattention/hyperactivity (SDQ) and pandemic‐related anxiety (PAS)	Parent	Change in emotional symptoms between March–April and April–May 2020 (acute) was not tested statistically in children and adolescents with ADHD and/or ASD. Change in conduct and inattention/hyperactivity problems between March–April and April–May 2020 (acute) was not tested statistically in children and adolescents with ADHD and/or ASD. Change in pandemic‐related anxiety between March–April and April–May 2020 (acute) was not tested statistically in children and adolescents with ADHD and/or ASD.
Houghton et al. (2022)	November 2018–April 2019	March 2020 July/August 2020	238 (76 ADHD, 36 ASD, 134 SLD, 19 other)	Depression symptoms (CDI:SR), internalising symptoms and externalising symptoms (SDQ)	YP	Depressive symptoms and internalising symptoms did not change significantly between pre‐pandemic and March 2020 (acute) or July/August 2020 (remission) in children and adolescents with neurodevelopmental conditions. Externalising symptoms did not change significantly between pre‐pandemic and March 2020 (acute) or July/August 2020 (remission) in children and adolescents with neurodevelopmental conditions. Depressive symptoms and internalising symptoms did not change significantly between March 2020 (acute) and July/August 2020 (remission) in children and adolescents with neurodevelopmental conditions. Externalising symptoms did not change significantly between March 2020 (acute) and July/August 2020 (remission) in children and adolescents with neurodevelopmental conditions.
Lugo‐Marín et al. (2021)	Pre‐COVID	May–July 2020	37	Internalising symptoms, externalising symptoms, depressive symptoms, anxiety symptoms, inattention/hyperactivity, oppositional defiant problems (CBCL)	Parent	Internalising, depressive and anxiety symptoms did not significantly change between pre‐pandemic and May–July 2020 (acute/remission) in autistic children and adolescents. Externalising symptoms, inattention/hyperactivity problems and oppositional defiant problems did not significantly change between pre‐pandemic and May–July 2020 (acute/remission) in autistic children and adolescents.
Prato et al. (2023)	N/A	June 2021–June 2022 (6 and 12 months after enrolment)	11	Depressive symptoms (CDI), anxiety symptoms (MASC), tic severity and impairment (YGTSS) and OCD symptoms (CY‐BOCS)	YP Clinician	Depressive and anxiety symptoms did not change significantly between enrolment (June 2021–June 2022; resurgence) 12‐month follow‐up in children and adolescents with tic disorder. Tic severity and impairment and OCD symptoms did not change significantly between enrolment (June 2021–June 2022; resurgence) 12‐month follow‐up in children and adolescents with tic disorder.
Raffagnato et al. (2021)	N/A	April–May 2020 August–September 2020	39	Internalising symptoms, externalising symptoms and post‐traumatic stress symptoms (CBCL and YSR)	YP Parent	Internalising symptoms significantly reduced between April–May (acute) and August–September 2020 (remission) in children and adolescents with affective, behavioural and neurodevelopmental conditions. Externalising symptoms did not change significantly between April–May (acute) and August–September 2020 (remission) in children and adolescents with affective, behavioural and neurodevelopmental conditions. Post‐traumatic stress symptoms significantly reduced between April–May (acute) and August–September 2020 (remission) in children and adolescents with affective, behavioural and neurodevelopmental conditions.
Sadeghi et al. (2022)	March 2019–March 2020	March–April 2020 April–May 2020 May 2020 May–June 2020 June–July 2020 July–September 2020 September 2020–January 2021 January–March 2021	96/97 (completed assessments for depressive and anxiety symptoms, respectively)	Depressive symptoms (MFQ) and anxiety symptoms (SCARED and SCARED‐short)	YP Parent	Depressive and anxiety symptoms did not change significantly between pre‐pandemic and during pandemic timepoints (March–April 2020 to January–March 2021) in adolescents with depression. Depressive and anxiety symptoms did not significantly change between March–April, April–May and May 2020 in adolescents with depression. Depressive and anxiety symptoms did not significantly change between acute (March–April, April–May and May 2020) and remission phases (May–June, June–July, July–September and September 2020–January 2021) in adolescents with depression. Depressive and anxiety symptoms did not significantly change between acute (March–April 2020, April–May and May 2020) and resurgence phases (January–March 2021) in adolescents with depression. Depressive and anxiety symptoms did not significantly change between acute (June–July and September 2020–January 2021) and resurgence phases (January–March 2021) in adolescents with depression.
Siracusano et al. (2021)	Pre‐COVID	May–July 2020	85 (33 2–5 years old, 52 6–18 years old)	Internalising symptoms and externalising symptoms (CBCL)	Parent	Internalising symptoms did not change between pre‐pandemic and May–July 2020 (acute/remission) in autistic children and adolescents. Externalising symptoms did not significantly change between pre‐pandemic and May–July 2020 (acute/remission) in autistic children and adolescents.
Summerton et al. (2023)	N/A	May 2020 May–June 2021	207	Depressive symptoms (SMFQ), anxiety symptoms (SCAS), irritability (ARI), inattention, hyperactivity/impulsivity and oppositional defiant symptoms (SNAP‐IV)	Parent	Change in depressive and anxiety symptoms between May 2020 (acute) and May–June 2021 (resurgence) in children and adolescents with ADHD was not tested statistically. Change in irritability, inattention, hyperactivity/impulsivity and oppositional defiant problems between May 2020 (acute) and May–June 2021 (resurgence) in children and adolescents with ADHD were not tested statistically.
Suzuki and Hiratani (2021)	April 2018–February 2020	October–December 2020	143	Irritability and hyperactivity (ABC‐J)	Parent	Irritability and hyperactivity problems significantly decreased between pre‐pandemic and October–December 2020 (remission) in children and adolescents with a neurodevelopmental condition.
Toseeb and Asbury (2023)	N/A	March–April 2020 April 2020–May 2020 May 2020–June 2020 September–October 2020	250	Depressive symptoms (RCADS) and anxiety symptoms (adapted RCADS)	Parent	Change in depressive and anxiety symptoms between March–April, April–May and May–June 2020 (acute) was not tested statistically in autistic children and adolescents and other SENDs. Between March–April (acute) and September–October 2020 (remission), depressive symptoms did not significantly change and anxiety symptoms significantly decreased in autistic children and adolescents or other SENDs.
Breaux et al. (2021) Fredrick et al. (2022) Dvorsky et al. (2022) Lewis et al. (2023)	September 2018–February 2020	May–June 2020 July–August 2020 October–November 2020 March–April/May 2021	118 236 (117 ADHD, 38 depression or anxiety)	Depressive symptoms, anxiety symptoms (RCADS), inattention, hyperactivity/impulsivity and oppositional defiant problems (VADRS) and pandemic‐related depressive and anxiety symptoms (CRISIS) Post‐traumatic stress symptoms (CATS)	YP Parent	Change in depressive and anxiety symptoms between pre‐pandemic and May–June (acute) or July–August 2020 (remission) were not tested statistically in adolescents with ADHD. Change in inattention, hyperactivity/impulsivity and oppositionality/defiance problems between pre‐pandemic and May–June (acute) or July–August 2020 (remission) were not tested statistically in adolescents with ADHD. Change in depressive and anxiety symptoms between May–June (acute) and July–August 2020 (remission) were not tested statistically in adolescents with ADHD. Change in inattention, hyperactivity/impulsivity and oppositionality/defiance problems between May–June (acute) and July–August 2020 (remission) were not tested statistically in adolescents with ADHD. Depressive and anxiety symptoms between May–June 2020 (acute) and March–April 2021 (resurgence) in adolescents with ADHD were not tested statistically. Change in pandemic‐related depressive and anxiety symptoms between May–June (acute) and July–August/October–November 2020 (remission) in adolescents with ADHD were not tested statistically. Change in pandemic‐related depressive and anxiety symptoms between July–August and October–November 2020 (remission) in adolescents with ADHD were not tested statistically. Post‐traumatic stress symptoms significantly reduced between May–June (acute) and July–August/October–November 2020 (remission) in adolescents with ADHD or internalising disorders. Post‐traumatic stress symptoms significantly reduced between May–June 2020 (acute) and March–May 2021 (resurgence) in adolescents with ADHD or internalising disorders. Post‐traumatic stress symptoms did not significantly change between July–August and October–November 2020 (remission) in adolescents with ADHD or internalising disorders. Post‐traumatic stress symptoms did not significantly change between July–August/October–November 2020 (remission) and March–May 2021 (resurgence) in adolescents with ADHD or internalising disorders.

### Risk of bias

Risk of bias was rated high in three studies, mainly due to small sample sizes, and moderate in 18 studies, mainly due to limitations in sampling and recruitment, survey delivery, attrition and confounding factors (Figure S2). Funnel plots (Figures S3–S5) and Egger's regression tests (Appendix S9) found limited evidence of publication bias.

### Pre‐ versus during pandemic mental health

#### Internalising symptoms

Ten studies assessed internalising symptoms at pre‐ and during pandemic timepoints (Table 1); one study reported a combined emotional and behavioural problem score (de Maat et al., 2023). Studies included autistic children and adolescents (four), children and adolescents with mixed neurodevelopmental conditions (two), ADHD (one), neuropsychiatric (one), MDD (one) and adolescents at high‐risk (one). Five studies had parent reported data, four had young people reported data, and one had parent and young people reported data. No studies reported change in overall parent or young people reported internalising symptoms in children and young people with neurodevelopmental or neuropsychiatric conditions pre‐pandemic versus acute/remission phases (Conti et al., 2020; De Giacomo et al., 2021; de Maat et al., 2023; Houghton et al., 2022; Lugo‐Marín et al., 2021; Siracusano et al., 2021).

For depressive symptoms, most studies found no significant change between pre‐pandemic and acute, remission and resurgence phases in autistic children and adolescents (Lugo‐Marín et al., 2021), or those with depression (Sadeghi et al., 2022), neuropsychiatric (Conti et al., 2020) or neurodevelopmental conditions (Houghton et al., 2022). One study found depressive symptoms increased significantly between pre‐pandemic and acute phase in high‐risk adolescents overall (Bouter et al., 2023). Of note, 19.3% saw an increase and 9.9% a decrease.

For anxiety symptoms, most studies found no significant change between pre‐pandemic and acute, remission and resurgence phases in autistic children and adolescents (Corbett, Muscatello, Klemencic, & Schwartzman, 2021; Lugo‐Marín et al., 2021) and those with depression (Sadeghi et al., 2022). One study found no change in the mid‐to‐late childhood group but significant increase in the early childhood group between pre‐pandemic and acute phase (Conti et al., 2020). Another study on high‐risk adolescents found a significant decrease in anxiety symptoms between pre‐pandemic and acute phase, where 8.5% of the sample improved and 8.5% worsened, respectively (Bouter et al., 2023).

**Figure 2:**
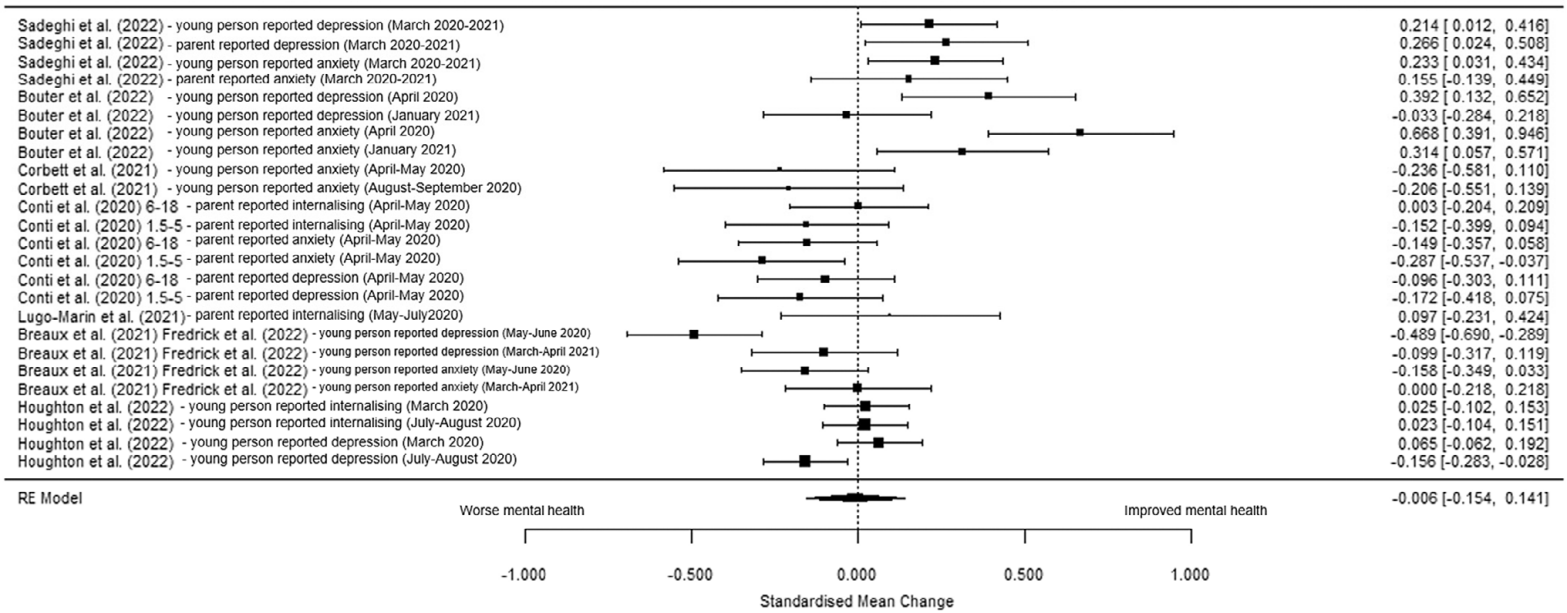
Forest plot of standardised mean change (SMC) in internalising symptoms pre‐ versus during pandemic

Seven studies were included in the multi‐level meta‐analysis (Table 3). There was no significant change in internalising symptoms during the pandemic compared to pre‐pandemic timepoints (SMC = −0.006 [95% CI: −0.154 to 0.141], *z* = −0.08, *p* = .933, *I* ^2^ = 81.05%). See Figure 2 for forest plot and Table S1 for distribution of variance across levels. Model comparison (Table S2) confirmed that the multi‐level model had a better fit than a conventional model, suggesting the data structure reflected true correlations between multiple effects within studies. Sensitivity analysis (Table 3) including de Maat et al.'s (2023) emotional and behavioural score was not significant (Figure S6). Subgroup analyses on only depressive, anxiety symptoms, young person reported data, age 10 years or over, or acute phase were not significant and did not reduce heterogeneity (Table 3; Figures S7–S11).

**Table 3:** Multi‐level meta‐analyses for standardised mean change in outcomes across pre‐ versus during pandemic mental health and during pandemic mental health

Research question	Outcome	Included studies	Effect estimate
Pre‐ versus during pandemic mental health	Internalising symptoms	Bouter et al. (2023) Breaux et al. (2021) Conti et al. (2020) Corbett et al. (2021) Houghton et al. (2022) Lugo‐Marín et al. (2021) Sadeghi et al. (2022)	SMC = −0.006 [95% CI: −0.154 to 0.141], *z* = −0.08, *p* = .933, *I* ^2^ = 81.05%
	Sensitivity: addition of emotional and behavioural problems	Bouter et al. (2023) Breaux et al. (2021) Conti et al. (2020) Corbett et al. (2021) de Maat et al. (2023) Houghton et al. (2022) Lugo‐Marín et al. (2021) Sadeghi et al. (2022)	SMC = −0.001 [95% CI: −0.136 to 0.134], *z* = −0.01, *p* = .992, *I* ^2^ = 79.36%
	Subgroup: only depressive symptoms	Bouter et al. (2023) Breaux et al. (2021) Corbett et al. (2021) Fredrick et al. (2022) Houghton et al. (2022) Sadeghi et al. (2022)	SMC = −0.023 [95% CI: −0.199 to 0.153], *z* = −0.25, *p* = .800, *I* ^2^ = 84.35%
	Subgroup: only anxiety symptoms	Bouter et al. (2023) Breaux et al. (2021) Conti et al. (2020) Fredrick et al. (2022) Houghton et al. (2022) Sadeghi et al. (2022)	SMC = −0.002 [95% CI: −0.237 to 0.234], z = −0.01, *p* = .988, *I* ^2^ = 83.06%
	Subgroup: only young people reported	Bouter et al. (2023) Breaux et al. (2021) Conti et al. (2020) Corbett et al. (2021) Fredrick et al. (2022) Sadeghi et al. (2022)	SMC = 0.032 [95% CI: −0.179 to 0.242], *z* = 0.29, *p* = .769, *I* ^2^ = 87.81%
	Externalising symptoms	Bouter et al. (2023) Breaux et al. (2021) Conti et al. (2020) Houghton et al. (2022) Lugo‐Marín et al. (2021)	SMC = 0.080 [95% CI: −0.120 to 0.279], *z* = 0.78, *p* = .434, *I* ^2^ = 84.43%
	Sensitivity: addition of emotional and behavioural problems	Bouter et al. (2023) Breaux et al. (2021) Conti et al. (2020) de Maat et al. (2023) Houghton et al. (2022) Lugo‐Marín et al. (2021)	SMC = 0.076 [95% CI: −0.095 to 0.247], *z* = 0.87, *p* = .384, *I* ^2^ = 81.34%
During pandemic mental health	Internalising symptoms	Breaux et al. (2021) Corbett et al. (2021) Houghton et al. (2022) Raffagnato et al. (2021) Sadeghi et al. (2022) Toseeb and Asbury (2023)	SMC = 0.106 [95% CI: −0.059 to 0.272], *z* = 1.26, *p* = .206, *I* ^2^ = 74.50%
	Subgroup: only young people reported	Breaux et al. (2021) Corbett et al. (2021) Houghton et al. (2022) Raffagnato et al. (2021) Sadeghi et al. (2022)	SMC = 0.100 [95% CI: −0.105 to 0.305], *z* = 0.96, *p* = .337, *I* ^2^ = 81.17%

#### Externalising symptoms

Nine studies assessed externalising symptoms at pre‐ and during pandemic timepoints (Table 1). Studies included autistic children and adolescents (three), children and adolescents with mixed neurodevelopmental conditions (three), ADHD (one), neuropsychiatric conditions (one) and adolescents at high‐risk (one). Six studies had parent reported data, two with young people reported data, and one with parent and young people reported data.

Overall, no studies reported change in overall parent or young people reported externalising symptoms in autistic children and young people and those with neurodevelopmental or neuropsychiatric conditions pre‐pandemic versus acute/remission phases (Conti et al., 2020; De Giacomo et al., 2021; de Maat et al., 2023; Houghton et al., 2022; Lugo‐Marín et al., 2021; Siracusano et al., 2021). Two studies found no change in oppositional defiant problems in high‐risk and autistic children and adolescents between pre‐pandemic and acute/remission phases (Bouter et al., 2023; Lugo‐Marín et al., 2021). To note, although Bouter et al. (2023) found overall no change in oppositional defiant problems, 5.6% and 3.8% of the sample worsened or improved, respectively. One study found irritability significantly decreased between pre‐pandemic and remission phase in children and adolescents with neurodevelopmental conditions (Suzuki & Hiratani, 2021).

There was mixed evidence for change in parent and young people reported inattention/hyperactivity problems. Two studies found no change between pre‐pandemic and acute/remission phases in autistic children and adolescents (Lugo‐Marín et al., 2021) or those with neuropsychiatric conditions (Conti et al., 2020), while another reported significant decrease in hyperactivity problems between pre‐pandemic and remissions phase in children and adolescents with neurodevelopmental conditions (Suzuki & Hiratani, 2021).

**Figure 3:**
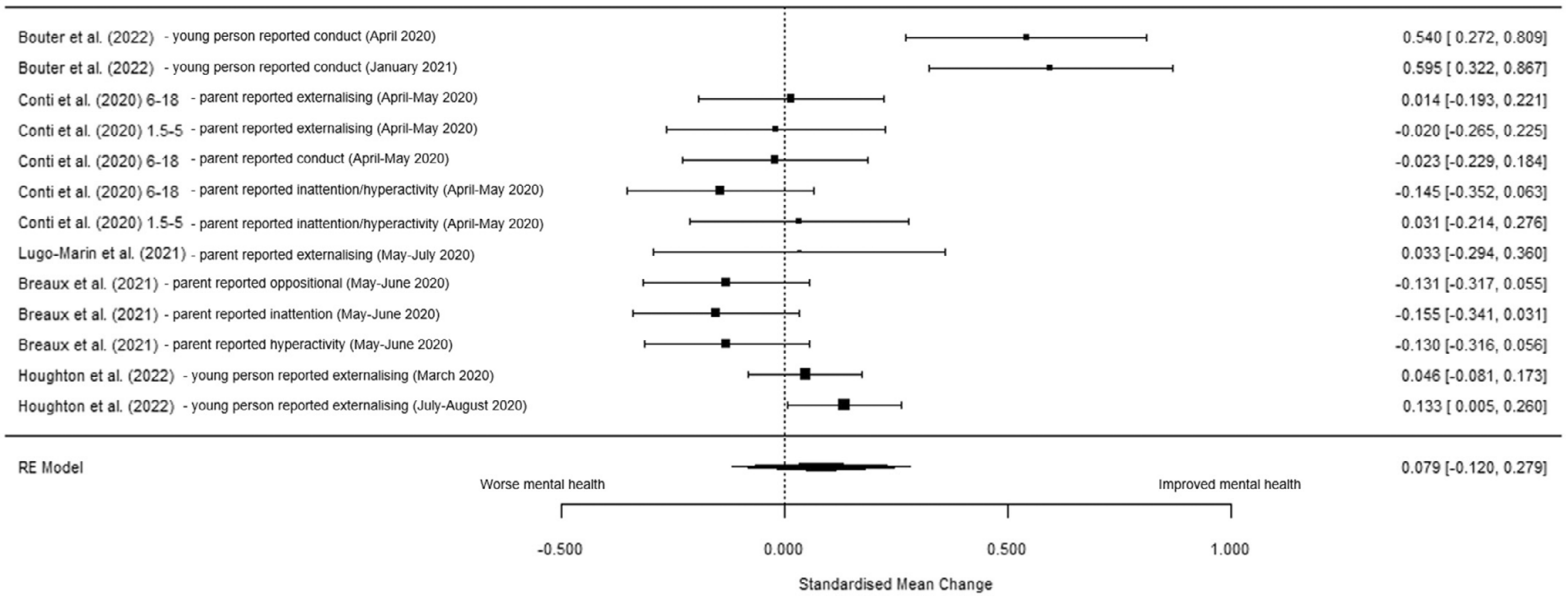
Forest plot of standardised mean change (SMC) in externalising symptoms pre‐ versus during pandemic

Five studies were included in the multi‐level meta‐analysis (Table 3). There was no significant change in externalising symptoms during the pandemic compared to pre‐pandemic timepoints (SMC = 0.080 [95% CI: −0.120 to 0.279], *z* = 0.78, *p* = .434, *I* ^2^ = 84.43%). See Figure 3 for forest plot and Table S3 for distribution of variance across levels. The multi‐level model fits better than the conventional (Table S4). Sensitivity analysis (Table 3) including de Maat et al. (2023) was not significant (Figure S12).

#### Other symptoms

Three studies assessed other symptoms at pre‐ and during pandemic timepoints, including obsessive–compulsive disorder (OCD), post‐traumatic stress, psychotic, tic symptoms and suicidality (Table 1). One study on children and young people with neuropsychiatric conditions reported a significant increase in parent reported OCD and post‐traumatic stress symptoms between pre‐pandemic and acute phase in the mid‐to‐late childhood group, but not in the early childhood group (Conti et al., 2020). Another study reported a reduction in young people reported psychotic symptoms and suicidality between pre‐pandemic and acute phase in high‐risk adolescents (Bouter et al., 2023). Hall, Marston et al. (2023) noted no significant change in clinician‐rated tics in children and young people with tic disorders between pre‐pandemic and acute/remission phases. No meta‐analysis was conducted due to the low number of studies.

### Mental health during the pandemic

#### Internalising symptoms

Twelve studies assessed overall internalising symptoms during the pandemic (Table 1): three during the acute phase, six during acute and remission, three during acute and resurgence, one during remission, one during remission and resurgence and one during resurgence (Figure S1). Two studies were from the same cohort and synthesised as one study (Breaux et al., 2021; Fredrick et al., 2022). Studies included autistic children and adolescents (three), children and adolescents with ADHD (three), those with mixed neurodevelopmental conditions (two), MDD (one), one tic disorder (one), mixed psychiatric and neurodevelopmental conditions (one), and adolescents at high‐risk (one). Five studies included parent and young people reported data, three with parent reported data, three with young people reported data, and one with young people and clinician reported data.

There was mixed evidence for parent and young people‐reported internalising, depressive and anxiety symptoms. During the remission phase, significant reduction in internalising symptoms was observed in autistic children and adolescents (Day, Gerber, McNair, Reicher, & Lerner, 2023). During the acute and resurgence phases, no change in depressive and anxiety symptoms was reported in adolescents with depression (Sadeghi et al., 2022) and in children and adolescents with tic disorder (Prato et al., 2023), respectively.

Between acute and remission phases, most studies reported no change in internalising, depressive and anxiety symptoms in autistic children and adolescents (Corbett et al., 2021; Toseeb & Asbury, 2023), or those with neurodevelopmental conditions (Houghton et al., 2022), other SENDs (Toseeb & Asbury, 2023) or depression (Sadeghi et al., 2022). Two studies reported significant reductions in internalising symptoms in children and adolescents with mixed psychiatric and neurodevelopmental conditions (Raffagnato et al., 2021) and anxiety symptoms in autistic children and other SENDs (Toseeb & Asbury, 2023), respectively.

Between acute and resurgence phases, one study found no change in depressive and anxiety symptoms in adolescents with depression (Sadeghi et al., 2022), while another study found significant increase in depressive symptoms but no change in anxiety in high‐risk adolescents (Bouter et al., 2023). The latter study also found variations within their sample, with 20.5% and 10% showing an increase and 7.3% and 4.4% a decrease in depressive and anxiety symptoms, respectively. Sadeghi et al. (2022) also reported no change in depressive and anxiety symptoms between remission and resurgence phases in adolescents with depression.

**Figure 4:**
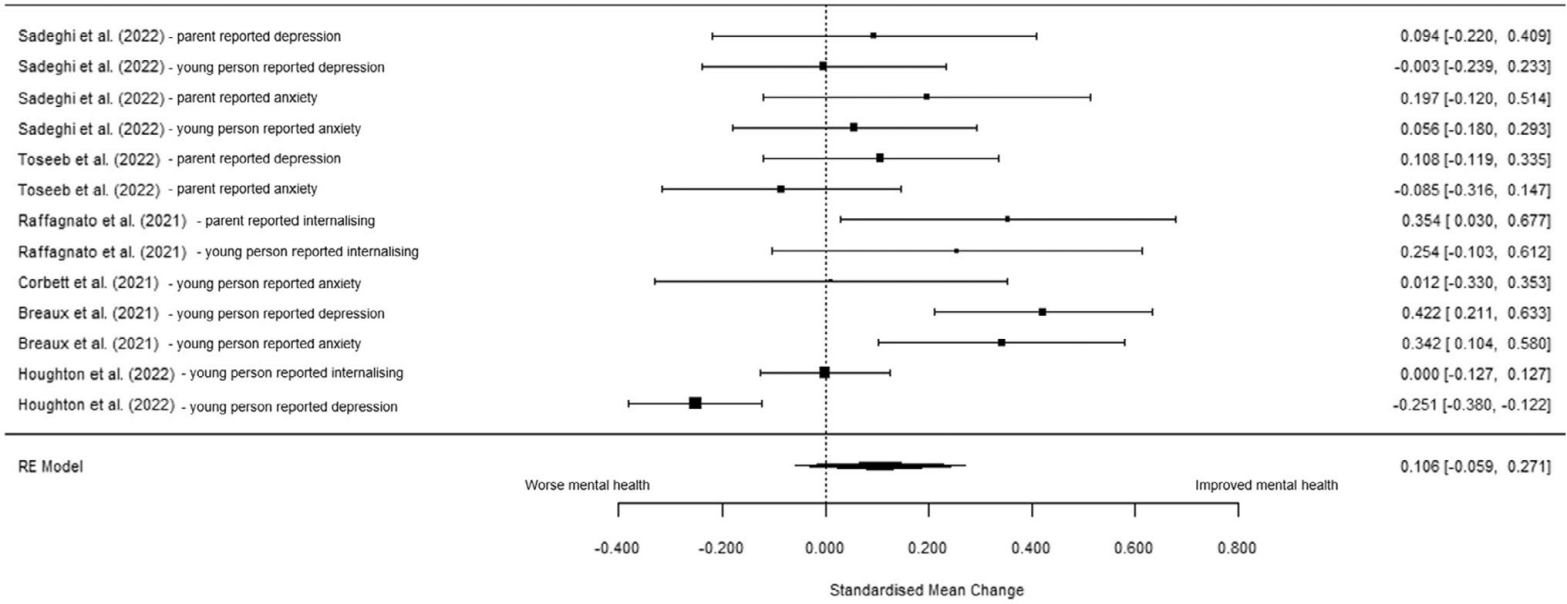
Forest plot of standardised mean change (SMC) in internalising symptoms between acute and remission pandemic phases

Six studies were included in the multi‐level meta‐analysis comparing internalising symptoms between acute and remission phases (Table 3). There was no significant change in internalising symptoms in remission compared to acute phase (SMC = 0.106 [95% CI: −0.059 to 0.272], *z* = 1.26, *p* = .206, *I* ^2^ = 74.50%). See Figure 4 for forest plot and Table S5 for distribution of variance across levels. Multi‐level model fitted better than the conventional (Table S6). Subgroup analysis (Table 3) on only young people reported data and age 10 years or above were non‐significant and did not reduce heterogeneity (Figures S13 and S14). No meta‐analyses between acute and resurgence or remission and resurgence phases were conducted due to insufficient number of studies.

#### Externalising symptoms

Six studies assessed overall externalising symptoms during the pandemic (Table 1): three presented data during acute and remission phase, two during acute and resurgence period, and one only in acute phase (Figure S1). One study with data in acute and remission phases did not report change so was not included in this synthesis (Houghton et al., 2022). One study included each of the following samples: autistic children and adolescents, adolescents with ADHD, mixed neurodevelopmental conditions, mixed emotional and neurodevelopmental conditions, and adolescents at high‐risk. Two studies had parent reported data, one with young person reported data, and two with parent and young person reported data.

Most studies reported no change in parent or young people reported externalising, inattention, hyperactivity/impulsivity and oppositional defiant problems in children and young people with psychiatric and neurodevelopmental conditions (Houghton et al., 2022; Raffagnato et al., 2021) and high‐risk adolescents (Bouter et al., 2023) across acute, remission and resurgence phases. However, although Bouter et al. (2023) found overall no change in oppositional defiant problems in high‐risk adolescents, 2.6% and 4.7% of the sample experienced an increase and decrease, respectively. No meta‐analysis was conducted due to an insufficient number of studies to make comparisons across phases.

#### Other symptoms

Six studies assessed other symptoms during the pandemic (Table 1): two studies during the acute and remission phase, one during the acute and resurgence period, one during the acute phase, one during resurgence and one across all three phases (Figure S1). Two studies reported significant reduction in post‐traumatic stress symptoms between acute and remission/resurgence phases in children and adolescents with psychiatric and neurodevelopmental conditions (Lewis et al., 2023; Raffagnato et al., 2021), and one found no change within/between remission and resurgence phases (Lewis et al., 2023). One study reported a significant decrease in psychotic symptoms but not suicidality between acute and resurgence phases in high‐risk adolescents (Bouter et al., 2023). Tic severity and impairment and OCD symptoms did not significantly change during the resurgence phase in children and adolescents with tic disorder (Prato et al., 2023). No meta‐analysis was conducted due to the insufficient number of studies.

#### Explanatory factors

Thirteen studies assessed a range of potential explanatory factors, identified by the narrative synthesis (Table 4).

**Table 4:** Explanatory factors of the longitudinal mental health impact of the COVID‐19 pandemic and reported direction of effect

Explanatory factor	Study	Effect
Clinical: diagnostic group (ADHD)	Houghton et al. (2022)	⇩
	Lewis et al. (2023)	⇔
Clinical: diagnostic group (ASD)	Hall, Partlett et al. (2023)	⇔
	Houghton et al. (2022)	⇔
	Toseeb and Asbury (2023)	⇧
Clinical: diagnostic group (emotional and behavioural disorders)	Conti et al. (2020)	⇧
Clinical: diagnostic group (internalising disorder)	Lewis et al. (2023)	⇔
Clinical: diagnostic group (neurodevelopmental conditions)	Conti et al. (2020)	⇧
Clinical: diagnostic group (neurological conditions)	Conti et al. (2020)	⇧
Clinical: diagnostic group (neuropsychiatric disorders)	Conti et al. (2020)	⇧
Clinical: diagnostic group (specific LDs)	Houghton et al. (2022)	⇔
Clinical: pre‐pandemic symptom severity (clinical or severe range)	Lugo‐Marín et al. (2021)	⇩
	Bouter et al. (2023)	⇩⇔
	Hall, Marston et al. (2023)	⇔
Clinical: treatment (CBT)	Sadeghi et al. (2022)	⇔
Clinical: treatment (medication status)	Dvorsky et al. (2022)	⇔
Clinical: pre‐pandemic emotion dysregulation	Breaux et al. (2021)	⇧
Sociodemographic: age	Conti et al. (2020)	⇧ in ≤5‐year‐olds
	Toseeb and Asbury (2023)	⇧ in older
	Lugo‐Marín et al. (2021)	⇔
	Hall, Marston et al. (2023)	⇔
Sociodemographic: sex/gender (female)	Sadeghi et al. (2022)	⇧
	Toseeb and Asbury (2023)	⇧
	Dvorsky et al. (2022)	⇧
	Lewis et al. (2023)	⇧⇔
	Fredrick et al. (2022)	⇔
	Hall, Marston et al. (2023)	⇔
Sociodemographic: financial hardship	Conti et al. (2020)	⇧
	Toseeb and Asbury (2023)	⇔
Sociodemographic: ethnicity	Toseeb and Asbury (2023)	⇔
	Dvorsky et al. (2022)	⇔
Pandemic‐related: COVID‐19 stress and impact	Lewis et al. (2023)	⇧
	Summerton et al. (2023)	⇧⇔
Pandemic‐related: education (mainstream school)	Toseeb and Asbury (2023)	⇧
Pandemic‐related: education (education, health and care plan (EHCP))	Toseeb and Asbury (2023)	⇔
Pandemic‐related: coping mechanisms (engagement in daily routines, activities and structure)	Dvorsky et al. (2022)	⇩
Pandemic‐related: parental mental health	de Maat et al. (2023)	⇔
Pandemic‐related: social connectedness	de Maat et al. (2023)	⇔

⇧⇔⇩ indicates that the explanatory factor was associated with increase, no change and decrease in mental health symptoms, respectively.

#### Clinical

Child clinical factors were noted in 10 studies. Five studies investigated diagnostic groups but there were inconsistencies in what diagnosis predicted worse outcomes. Conti et al. (2020) reported neurodevelopmental disorder‐related problems were worse in children with emotional and behavioural disorders and anxiety symptoms were worse in those with neurological conditions in the early childhood group between pre‐pandemic and acute phase. However, in the mid‐to‐late childhood group, emotional and behavioural problems were worse in those with emotional and behavioural problems and externalising symptoms were worse in those with neurodevelopmental conditions (Conti et al., 2020). Being autistic was associated with worse depressive and anxiety symptoms than those with other SENDs between acute and remission phases (Toseeb & Asbury, 2023), but not with tic severity and impairment in children and adolescents with tic disorder between pre‐pandemic and acute/remission phases (Hall, Marston et al., 2023). Having ADHD was found to be protective for externalising symptoms compared to those with specific LDs or autistic youth between pre‐pandemic and remission phases (Houghton et al., 2022). Lewis et al. (2023) found ADHD or internalising disorder diagnostic status did not impact post‐traumatic stress symptom trajectories in adolescents with ADHD or internalising disorders.

Four studies investigated other pre‐existing clinical characteristics: three looked at pre‐pandemic symptom severity and one on pre‐pandemic emotion regulation. High symptom severity was mostly associated with better outcomes during the pandemic; one study found that high‐risk adolescents who scored in the clinical range pre‐pandemic had the largest decrease in depressive and anxiety symptoms, oppositional defiant problems and psychotic symptoms in high‐risk adolescents across pre‐pandemic and acute/resurgence phases, while those in the ‘normal’ range saw small increases only (Bouter et al., 2023). Similarly, Lugo‐Marín et al. (2021) reported that autistic children with increased pre‐pandemic symptom severity reported significantly reduced internalising, anxiety and depressive symptoms, and oppositional defiant problems between pre‐pandemic and acute/remission phases compared to those with less symptom severity. However, two studies found pre‐pandemic symptoms did not influence suicidality (Bouter et al., 2023) or tic severity and impairment (Hall, Marston et al., 2023) across pre‐pandemic and pandemic phases. Breaux et al. (2021) found inattention and hyperactivity/impulsivity problems were significantly increased in adolescents with ADHD with poor pre‐pandemic emotion regulation skills across pre‐pandemic and acute/remission phases (Breaux et al., 2021).

Two studies looked at treatment; receiving treatment during pandemic, such as cognitive behavioural therapy or medication, did not predict changes in depressive and anxiety symptoms in adolescents with depression or ADHD during pandemic phases (Dvorsky et al., 2022; Sadeghi et al., 2022).

#### Sociodemographic

Eight studies investigated sociodemographic factors as explanatory factors. The evidence for age as a predictor of outcomes was mixed. Two studies reported that emotional and behavioural problems were worse in older autistic children and adolescents (Toseeb & Asbury, 2023) and older adolescents with neuropsychiatric conditions in the mid‐to‐late childhood/adolescence group (Conti et al., 2020) across pre‐pandemic and acute/remission phases. However, two studies on autistic children and adolescents (Lugo‐Marín et al., 2021) and those with tic disorders (Hall, Marston et al., 2023) did not find age‐varying effects.

Most evidence suggested that being female predicted worse outcomes. Being female was associated with worse depressive and anxiety symptoms across pre‐pandemic, acute and remission phases in autistic adolescents (Toseeb & Asbury, 2023) or those with depression (Sadeghi et al., 2022) or ADHD (Dvorsky et al., 2022). Lewis et al. (2023) also reported that female adolescents with ADHD or internalising disorder were significantly more likely to have severe fluctuating post‐traumatic stress symptoms and significantly less likely to remain in the non‐clinical range than males between acute and resurgence phases. However, two studies reported no impact of sex on depressive and anxiety symptoms in adolescents with ADHD (Fredrick et al., 2022), or tic severity and impairment in adolescents with tic disorder (Hall, Marston et al., 2023), respectively.

Other studies reported mixed‐to‐no evidence for financial hardship and ethnicity. Conti et al. (2020) noted internalising, post‐traumatic stress and obsessive–compulsive symptoms were worse in children with neuropsychiatric disorders with increased financial hardship between pre‐pandemic and acute phase. However, Toseeb and Asbury (2023) found no impact of income or ethnicity on depressive and anxiety symptoms in autistic children and adolescents and other SENDs. Dvorsky et al. (2022) reported that race/ethnicity did not predict pandemic‐related depressive and anxiety symptoms in adolescents with ADHD across acute/remission phases.

#### Pandemic‐related

Pandemic‐related factors were highlighted in five studies. Summerton et al. (2023) reported COVID‐19 stress in the acute phase was significantly associated with increased hyperactivity/impulsivity problems in resurgence phase, but not with depressive and anxiety symptoms, irritability, inattention or oppositional defiant problems in children and adolescents with ADHD. Lewis et al. (2023) found adolescents with ADHD or internalising disorders who reported higher COVID‐19 impact were significantly more likely to have moderate‐to‐clinical post‐traumatic stress symptoms across acute and resurgence phases.

There was some evidence for effects of education and engagement coping mechanisms. Toseeb and Asbury (2023) noted that depressive symptoms, but not anxiety, were worse in autistic and other SEND youth who were in mainstream school during the pandemic compared to those with alternate provision. However, receiving an education, health and care plan (EHCP) did not impact depressive and anxiety symptoms (Toseeb & Asbury, 2023). Engagement in daily routines, activities and structure in the acute phase significantly predicted pandemic‐related depressive and anxiety symptoms in the remission phase in adolescents with ADHD (Dvorsky et al., 2022).

There was no evidence for other parent and social factors. Parental mental health and social connectedness were unrelated to changes in emotional and behavioural problems in autistic children and adolescents between pre‐pandemic and resurgence phase (de Maat et al., 2023).

## Discussion

### Summary of findings

We conducted a comprehensive systematic review investigating mental health changes in children and young people with pre‐existing mental health and neurodevelopmental conditions pre‐ versus during and across pandemic phases. Contrary to our theoretical rationale that this clinical group would have demonstrated increased mental health symptoms, the heterogeneous results suggest that the impact of the pandemic on those with pre‐existing conditions may be more varied and complex. The narrative synthesis and multi‐level meta‐analysis indicated no overall change in internalising, externalising and other symptoms pre‐ versus during the pandemic and across pandemic phases. Subgroup analyses were conducted where possible to investigate precise estimates on mental health changes by characteristics and parse heterogeneity but were non‐significant and limited due to number of studies. Analysis of individual studies found variation in effect, suggesting that the pandemic may have had varied mental health impact; specific studies provided some evidence of individual increases in internalising, post‐traumatic stress and obsessive–compulsive symptoms and reductions in internalising, externalising, post‐traumatic stress and psychotic symptoms during the pandemic. Within‐study variability was also seen; in some studies that showed overall no change, distinct subsamples with differential outcomes were identified. This may suggest that examining mental health changes through overall means and pooled effects may not capture divergent effects within samples, which are often heterogeneous.

Our narrative synthesis did not identify explanatory factors that ubiquitously explained the differential effects. Some studies suggested that factors such as diagnostic group, baseline symptom severity, age and sex/gender may play a role in the variation of pandemic effects seen within individual studies. These and other unmeasured factors may partly explain the observed heterogeneity. This further adds to the complexity of results and suggests that our rationale may be more nuanced, where children and young people with pre‐existing conditions' mental health may be differentially impacted by the pandemic due to a combination of explanatory factors.

### Comparison with other studies

Our findings align with previous COVID‐related systematic reviews on children and young people, which convey a highly varied picture of pandemic‐related mental health outcomes. A meta‐analysis reported significant worsening of internalising and externalising symptoms during versus pre‐pandemic (Bussières et al., 2021). However, only two of the included studies recruited children with pre‐existing conditions, and the meta‐analysis combined prospective and retrospective studies, where recall biases may offset a true time dependency on symptom changes. Another systematic review and meta‐analysis of 51 studies reported no significant change in anxiety symptoms and inattention/hyperactivity problems, mixed evidence of change in internalising and depressive symptoms, and significant improvement in conduct problems pre‐ versus during pandemic in children and young people (Newlove‐Delgado et al., 2023). However, significant increases in internalising symptoms and depressive symptoms and reduction in conduct problems were a result of pooling the effects of two to four studies per meta‐analysis. Narrative syntheses also highlighted variation among individual studies, as they reported increases, reductions and no changes (Ahmed et al., 2023; Dessain et al., 2023; Newlove‐Delgado et al., 2023; Panchal et al., 2023). Our findings are further echoed in qualitative studies (Asbury & Toseeb, 2023; Lenoir & Wong, 2023; McKinlay, May, Dawes, Fancourt, & Burton, 2022; Pearcey et al., 2023) and lived experience (Ching, Parlatini, Downs, & Simonoff, 2024; Ford, Newlove‐Delgado, Sabu, & Russell, 2024), illustrating a complex landscape of positive and challenging experiences.

Our narrative synthesis revealed several sociodemographic, clinical and pandemic‐related factors that may explain the differential and variable effects seen in the literature. It has been reported across cohort studies that those with neurodevelopmental conditions had the worst mental health during the pandemic (e.g. Guzman Holst et al., 2023; Parlatini et al., 2023). However, this association was inconsistently reported (Conti et al., 2020; Hall, Partlett et al., 2023) and may point to the low number of studies that investigated differential effects across diagnoses. At present, the data available prevents a meta‐analysis as studies tended to lump diagnostic groups together and report average effects. Additionally, it may highlight that pre‐existing mental health or neurodevelopmental conditions may be a general risk factor for poorer mental health. Most studies that included controls without pre‐existing conditions reported significantly worse mental health outcomes in clinical groups (Bouter et al., 2023; Breaux et al., 2021; Day et al., 2023; de Maat et al., 2023; Dvorsky et al., 2022; Houghton et al., 2022; Sadeghi et al., 2022), indicating that mental health trajectories in those with pre‐existing conditions may be distinct to the general population.

Our non‐significant meta‐analytic results do not reflect service data, which suggests mental health problems increased significantly for children and young people. In the UK, an initial decline in referrals to child and adolescent mental health services was observed at the beginning of the pandemic, followed by a sharp increase in autumn 2020 after school reopening (McNicholas et al., 2021). Examination of global paediatric emergency department mental health presentations showed a fluctuating increase in complexity during the pandemic (Wong et al., 2023). One possible explanation of divergent findings between our meta‐analysis and existing service data may be that our findings did not fully capture the longitudinal impact of the pandemic as only data in 2020 could be meta‐analysed. Another reason may be that our included data was unable to capture granular levels of changes that service data does. Service data trajectories were analysed using data of shorter intervals (e.g. per month) which captured outcome changes more precisely than our data, which were organised into heterogenous pandemic phases because study timepoints spanned multiple months. Those attending services may represent a subgroup of children and young people with pre‐existing conditions who deteriorated during pandemic, which our included studies have highlighted only make up a proportion of their samples. Mental health outcomes should be mapped onto service use data to better explore this discrepancy.

### Strengths and limitations

Overall, our study considered a diverse range of diagnoses and symptoms, capturing a wide‐ranging picture of the mental health impact of the pandemic. We synthesised longitudinal studies from pre‐pandemic to mid‐2021, which allowed for more detailed and long‐term comparisons of mental health changes. Categorisation of pandemic timepoints into phases facilitated meaningful comparisons of mental health changes within the pandemic. Multi‐level meta‐analyses allowed the inclusion of multiple study effects, modelled the hierarchical data structure, provided more precise effect size estimates and standard errors, and facilitated exploration of within‐ and between‐study heterogeneity.

The limitations of the literature and our systematic review should be considered. Most included studies did not report data attrition and those that did found study drop out ranges between 23%–70%. Bouter et al. (2023) found that children who were male, immigrants and from lowest income households and educational attainment were significantly more likely to not respond to follow‐up. Analysis methods used to explore explanatory factors were inconsistent where some studies used simple mean comparisons of stratified data by characteristic/group while other studies adjusted for multiple covariates. Moreover, studies did not consistently adjust for the same confounding variables, including key characteristics such as age, sex/gender, ethnicity and socioeconomic position. We could not meta‐analyse all eligible studies as five studies had missing data. Mental health changes between acute/remission and resurgence phases and many subgroup analyses could not be meta‐analysed due to insufficient numbers, and most of the studies were underpowered (e.g. 76% had <200 participants). These factors limited our ability to carry out further analysis of change and understand reasons for heterogeneity. Meta‐regressions accounting for geographical setting were also not possible due to low variation in target variables in our included studies. Included studies treated time periods that may contain multiple pandemic‐related events as one ‘single’ event, which restricted the ability to distinguish the impact of events, such as school closures and social restrictions, versus natural fluctuations in mental health. By the nature of the pandemic, many studies were opportunistic and did not include the range of data that would allow systematic exploration of explanatory factors; and there was a lack of consistency in what explanatory factors were assessed across studies. Moreover, our review only included studies where at least a subsample was clinically diagnosed, attended services or endorsed clinically significant symptoms, thus we may have missed children and young people with pre‐existing but undiagnosed conditions or who did not receive support.

### Future directions

The wide variability identified across studies suggests there may be important explanatory factors that play a role in the impact of the pandemic. These factors need to be further investigated to understand the mechanisms of risk and protection that the pandemic may engender. We summarised our research recommendations in Table 5. Research on pandemic effects on mental health should go beyond overall change and investigate subgroup changes to capture divergent impacts. Investigation of data post‐2021 is needed to expand our understanding of the pandemic's long‐term impact on mental health outcomes and longitudinal trajectories, such as later pandemic restrictions and its impact even after being lifted. Both quantitative and qualitative methodology may better explore reasons for heterogeneous experiences of the pandemic. Understanding what factors may relate to better or worse mental health outcomes in this population is crucial for clinical services and policy makers to provide targeted service provisions to those most affected and for service planning in the event of future pandemics/epidemics.

**Table 5:** Key research recommendations for future research on the longitudinal impact of the COVID‐19 pandemic on the mental health of children and young people

Recommendations
Consistently report key characteristics that may explain variability in mental health outcomes (e.g. age, sex/gender, ethnicity, social deprivation, diagnosis). This ensures that key confounding variables are accounted for in analysis and researchers can extract data by these potential explanatory factors. Explore beyond mean change and investigate subgroup differences. Looking at data and differences in mental health within samples will allow for comparisons between subgroups and exploration of potentially heterogeneous effects. Investigate potential differential effects and explanatory factors using robust statistical methods (e.g. address missing data, adjust for covariates). This will account for potential methodological reasons for heterogeneity. Analyse longitudinal data beyond 2021 to expand our understanding of the pandemic's long‐term impact. The data included in our systematic review is limited to only early‐to‐mid 2021, while pandemic‐related restrictions were still implemented beyond this date in certain countries. Furthermore, there could be longer‐term impacts even after restrictions have ended. Explore longitudinal mental health trajectories across diagnostic groups and general population. Further parsing of heterogeneity by clinical characteristics and other known risk factors may have important clinical implications. Map mental health outcomes onto service use data to inform service provision. As research and clinical data may measure different elements of mental health outcomes, it would be important to understand how mental health changes during the pandemic are reflected in service use data. Use quantitative and qualitative methods to explore contextual and mechanistic explanations for effect heterogeneity. Rich qualitative accounts of mental health and individual experiences may complement quantitative data on explanatory factors to improve understanding of reasons for divergent mental health changes.

## Conclusion

Our narrative synthesis and meta‐analyses found no significant overall changes in mental health symptoms pre‐ versus during pandemic and across pandemic phases. However, evidence from individual studies demonstrated varied effects that were averaged into null findings. Narrative synthesis identified potential explanatory factors that may explain which subgroups of children and young people did better or worse during the pandemic. Further longitudinal research needs to comprehensively investigate explanatory factors that may contribute to differential effects in this population. Understanding this is vital to support the management of long‐term mental health consequences and to guide effective responses to future pandemics.

**Key points**

- Existing systematic reviews have found mixed effects of the COVID‐19 pandemic on the mental health of children and young people, but the studies included were mostly cross‐sectional and/or on general population samples.
- This is the first systematic review to assess the longitudinal impact of the pandemic on the mental health of children and young people with pre‐existing mental health and neurodevelopmental conditions and related explanatory factors.
- Narrative synthesis found large heterogeneity in individual study findings, suggesting varied and potentially divergent effects.
- Multi‐level meta‐analyses on subsample of studies indicated no significant changes in internalising and externalising symptoms pre‐ versus during or across pandemic phases.
- Future longitudinal research is warranted to assess longer‐term impacts and understand differential effects on mental health to inform policy and support targeted mental health service provisions.

## Supporting information


**Appendix S1.** Eligibility criteria.
**Appendix S2.** Mental health outcomes included in the study.
**Appendix S3.** Search strategy.
**Appendix S4.** Further information on data extraction.
**Appendix S5.** Risk of bias indicators.
**Appendix S6.** Further details of statistical analysis.
**Appendix S7.** Further information on meta‐analyses.
**Appendix S8.** Further information on narrative synthesis.
**Appendix S9.** Egger's regression tests of funnel plot asymmetry.
**Figure S1.** COVID‐19 timeline and included studies.
**Figure S2.** Traffic light plot of risk of bias of included studies.
**Figure S3.** Funnel plot of studies assessing internalising symptoms pre‐ versus during pandemic.
**Figure S4.** Funnel plot of studies assessing externalising symptoms pre‐ versus during pandemic.
**Figure S5.** Funnel plot of studies assessing internalising symptoms during pandemic.
**Figure S6.** Forest plot of sensitivity analysis of the inclusion of a study with a combined emotional and behavioural problem score for internalising symptoms pre‐ versus during pandemic.
**Figure S7.** Forest plot of subgroup analysis by depressive symptoms for internalising symptoms pre‐versus during pandemic.
**Figure S8.** Forest plot of subgroup analysis by anxiety symptoms for internalising symptoms pre‐ versus during pandemic.
**Figure S9.** Forest plot of subgroup analysis by young people reported data for internalising symptoms pre‐ versus during pandemic.
**Figure S10.** Forest plot of subgroup analysis by age 10 years or over for internalising symptoms pre‐ versus during pandemic.
**Figure S11.** Forest plot of subgroup analysis by acute phase for internalising symptoms during pandemic.
**Figure S12.** Forest plot of sensitivity analysis of the inclusion of a study with a combined emotional and behavioural problem score for externalising symptoms pre‐ versus during pandemic.
**Figure S13.** Forest plot of subgroup analysis by young people reported data for internalising symptoms between acute and remission phases.
**Figure S14.** Forest plot of subgroup analysis by yage 10 years or above for internalising symptoms between acute and remission phases.
**Table S1.** Multi‐level *I* ^2^ distribution for internalising symptoms between pre‐ and during pandemic.
**Table S2.** Meta‐analysis model comparison for internalising symptoms between pre‐ and during pandemic.
**Table S3.** Multi‐level *I* ^2^ distribution for externalising symptoms between pre‐ and during pandemic.
**Table S4.** Meta‐analysis model comparison for externalising symptoms between pre‐ and during pandemic.
**Table S5.** Multi‐level *I* ^2^ distribution for internalising symptoms between during pandemic timepoints.
**Table S6.** Meta‐analysis model comparison for internalising symptoms between during pandemic timepoints.

**Data S1.** PRISMA checklist.

## Data Availability

Data used for analyses and R codes can be provided at request.
