# Supplementary material for: Research Review: The impact of the COVID‐19 pandemic on the mental health of children and young people with pre‐existing mental health and neurodevelopmental conditions – a systematic review and meta‐analysis of longitudinal studies
Source: J Child Psychol Psychiatry. 2025 Jan 30;66(8):1274–98. doi: 10.1111/jcpp.14117 (PMC12267672; doi:10.1111/jcpp.14117)
Supplement: Supplementary file 1 — Appendix S1. Eligibility criteria. Appendix S2. Mental health outcomes included in the study. Appendix S3. Search strategy. Appendix S4. Further information on data extraction. Appendix S5. Risk of bias indicators. Appendix S6. Further details of statistical analysis. Appendix S7. Further information on meta‐analyses. Appendix S8. Further information on narrative synthesis. Appendix S9. Egger's regression tests of funnel plot asymmetry. Figure S1. COVID‐19 timeline and included studies. Figure S2. Traffic light plot of risk of bias of included studies. Figure S3. Funnel plot of studies assessing internalising symptoms pre‐ versus during pandemic. Figure S4. Funnel plot of studies assessing externalising symptoms pre‐ versus during pandemic. Figure S5. Funnel plot of studies assessing internalising symptoms during pandemic. Figure S6. Forest plot of sensitivity analysis of the inclusion of a study with a combined emotional and behavioural problem score for internalising symptoms pre‐ versus during pandemic. Figure S7. Forest plot of subgroup analysis by depressive symptoms for internalising symptoms pre‐versus during pandemic. Figure S8. Forest plot of subgroup analysis by anxiety symptoms for internalising symptoms pre‐ versus during pandemic. Figure S9. Forest plot of subgroup analysis by young people reported data for internalising symptoms pre‐ versus during pandemic. Figure S10. Forest plot of subgroup analysis by age 10 years or over for internalising symptoms pre‐ versus during pandemic. Figure S11. Forest plot of subgroup analysis by acute phase for internalising symptoms during pandemic. Figure S12. Forest plot of sensitivity analysis of the inclusion of a study with a combined emotional and behavioural problem score for externalising symptoms pre‐ versus during pandemic. Figure S13. Forest plot of subgroup analysis by young people reported data for internalising symptoms between acute and remission phases. Figure S14. Forest plot of subgroup analysis by yag [file JCPP-66-1274-s001.docx]

# The impact of the COVID-19 pandemic on the mental health of children and young people with pre-existing mental health and neurodevelopmental conditions: a systematic review and meta-analysis of longitudinal studies

Ching et al.

## Supporting Information

**Appendix S1** Eligibility criteria.

Longitudinal studies reporting mental health outcomes in children and young people (≤18 years old at follow-up) with pre-existing mental health and/or neurodevelopmental conditions were included.

Studies were deemed eligible if children and young people:

- Were clinically diagnosed according to the Diagnostic and Statistical Manual of Mental Disorders (American Psychiatric Association, 2013) or International Classification of Diseases (World Health Organization, 2019). We considered clinical diagnoses, made by a clinician using validated clinical interviews. Self-reported clinical diagnoses (in the absence of the administration of clinical interview or assessment) were also permitted to maximise the number of the included papers, provided that studies aimed at recruiting clinical groups of children and young people.
- Scored above clinical threshold on validated measures. Example validated measures included the Strengths and Difficulties Questionnaire (SDQ). If at least 50% of the sample scored above the clinical threshold of the specific measure, they would be included as their symptoms would be classified as clinically significant. OR
- Attended mental health services pre-pandemic. Children and young people who were recruited from mental health services would be eligible to be included in the review as their presenting difficulties warranted acceptance into the service (which can be assumed to be high considering typically high thresholds for mental health services). This was used as a proxy for pre-existing mental health and/or neurodevelopmental conditions.

Studies reporting data on mixed children and adult samples were eligible if at least 50% of the sample were ≤18 and child data could be extracted. We included studies that compared outcomes pre- versus during pandemic, and/or between during pandemic timepoints, to examine how mental health changed during the pandemic compared to pre-pandemic and the potentially variable and temporal changes during the pandemic.

**Appendix S2** Mental health outcomes included in the study.

Mental health outcomes were aggregated into three broad symptom categories based on ICD-11 criteria: internalising symptoms, including depressive and anxiety symptoms; externalising symptoms, including conduct, oppositional defiant, inattention, and hyperactivity problems; and other symptoms, including psychotic, post-traumatic stress, obsessive compulsive, and tic symptoms. Only continuous mental health outcomes were extracted to support the assessment of changes over time. Only outcomes measured with standardised questionnaires were considered, such as the Strengths and Difficulties Questionnaire (SDQ; Goodman & Goodman, 2009), Revised Children Anxiety and Depression scale (RCADS; Chorpita et al., 2005), Child Behaviour Checklist (CBCL; Achenbach, 2001), Yale Global Tic Severity Scale (YGTSS; Leckman et al., 1989), and Coronavirus Health Impact Survey (CRISIS; Nikolaidis et al., 2020).

**Appendix S3** Search strategy.

1. COVID OR SARS-Cov OR coronavirus
2. child* OR adolesc* OR teen* OR “young people” OR “young person” OR youth
3. “mental disorder” OR “mental illness” OR “emotional difficulties” OR “emotional disorder” OR behavio* OR conduct OR neurodevelopmental OR autis* OR “attention deficit” OR adhd
4. observational OR longitudinal OR cross-sectional OR cohort OR case-control
5. 1 AND 2 AND 3 AND 4

Note: The search was conducted by searching in title, abstract, and keywords fields. Date filters were used to identify studies published since 2020 as no studies published before this would have assessed the mental health impact of the pandemic.

**Appendix S4** Further information on data extraction.

Data extracted included study characteristics (i.e., first author, publication year), sample characteristics (i.e., sample, setting, diagnostic criteria, sample size, age, gender, ethnicity), relevant mental health outcomes and assessment timepoints (i.e., outcome measure(s), timepoints, follow-up duration, attrition, informant(s)), and summary of findings (including potential explanatory factors of effect). Statistics extracted included mean and standard deviation (SD) of outcomes at each timepoint and correlations of outcomes between timepoints. Authors of papers were contacted about missing data at least three times; no response was noted as not reported. Data extraction was conducted by BCFC with ~75% of studies checked by SZ and TP for accuracy.

OxCGRT score is rated in the system out of 100, where higher scores indicated stricter measures (Hale et al., 2021). Restriction levels of each study timepoint were assessed by using the average of the OxCGRT score within the specific follow-up timepoint.

See below for the template data extraction sheet that was completed with available data that was presented in each paper and additional provided information by study authors:

| Author (year) | Country | Baseline sample | Baseline N | Diagnostic criteria | Baseline mean age (SD), range | Gender | Ethnicity | Pre-COVID wave | During COVID wave(s) | Duration of follow-up | Attrition at follow-up | OxCGRT score | Relevant outcome(s) (measure(s)) | Informant(s) | Extractable main statistics | Key findings | Explanatory factors | Meta-analysis | Notes |
| --- | --- | --- | --- | --- | --- | --- | --- | --- | --- | --- | --- | --- | --- | --- | --- | --- | --- | --- | --- |
| First author et al. and the year of publication. | Country study is based at. | Clinical group (e.g. children with ADHD, ASD, depression). Setting (e.g. school, mental health service, cohort study). | Number of participants at baseline of interested sample. | Method of eligibility of participants were assessed (e.g. self-report diagnosis, clinician administered diagnostic interview, meeting clinical threshold of a validated measure). | Descriptive statistics of age in the interested sample (or whole cohort if delineation is not possible). | Distribution of gender in the interested sample (or whole cohort if delineation is not possible), such as N or percentage. | Distribution of ethnicity in the interested sample (or whole cohort if delineation is not possible), such as N or percentage. | Time frame of the pre-COVID-19 timepoint. | Time frame(s) of the during COVID-19 timepoint(s). | Approximate number of months between first and final timepoints. | Descriptive statistics of attrition or retention of participants in the interested sample (or whole cohort if delineation is not possible), such as percentage. | Based on OxCGRT (the highest containment and health index (CHI) during the follow-up period. However, also considered the time/length of restrictions, e.g. if 85 for one month but 75 for 4 months, used 75). Use the top spectrum of the CHI to be overly inclusive. | Mental health symptoms and the scales (standardised measures) used to assess them. | Individual who completed the measures (i.e. parent, young person, clinician). | Inferential statistics of analyses investigating the changes in mental health symptoms. | Narrative summary of the key findings from the study. | Narrative summary of potential explanatory factors that may explain changes in mental health symptoms. | Eligibility for inclusion in meta-analysis. | Any other details to note. |


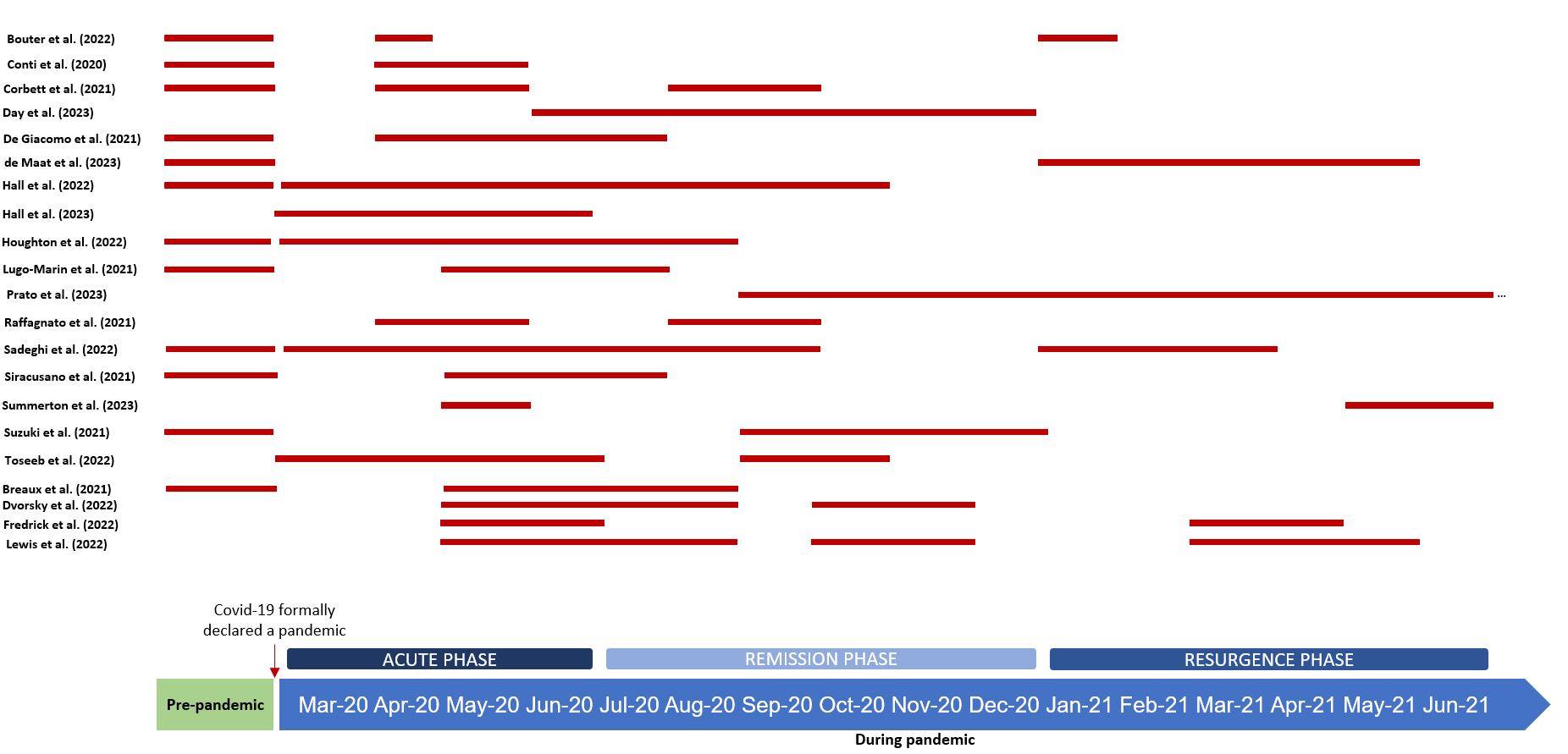


**Figure S1.** Timeline of follow-ups in included studies.

**Appendix S5** Risk of bias indicators.

Three study quality appraisal and risk of bias assessment scales (i.e., Newcastle-Ottawa Scale for Non-randomised Studies, National Institute of Health Quality Assessment Tool for Observational Cohort and Cross-sectional Studies, and Critical Appraisal Skills Programme Checklist for Cohort Studies) were reviewed to develop a list of risk of bias indicators to suit our research questions and included studies:

1. Sampling and recruitment - Were sampling methods used to achieve a representative sample of the population being studied, e.g., use of quota sampling or probability sampling to reflect demographics of wider population?
2. Sample size - Was a sample size justification, power description, or variance and effect estimates provided?
3. Survey delivery - Was survey delivery mode consistent across data collection timepoints, e.g., in-person or online?
4. Outcome assessment - Were measures of mental health outcomes collected more than 12-months apart from each other?
5. Attrition - Was attrition/loss to follow-up after baseline 20% or less and were appropriate strategies used to address attrition, e.g., analysis of whether attrition occurred at random and if not, strategies for selective attrition, such as weighting?
6. Confounding - Were confounding factors considered in the design and/or analysis, e.g., adjusted statistically?

Overall risk of bias: A rating of high, moderate/unclear, low, and not applicable were given for each indicator, where lower ratings indicated lower risk of bias. An overall quality rating was given for each study, where a study would be rated an overall high risk of bias if at least two indicators were rated high without any rated low, moderate if there were any indicators rated medium or high without meeting an overall high risk of bias, and low if all indicators were rated low.

**Appendix S6** Further details of statistical analysis.

Meta-analyses were performed where ≥5 studies reported the same outcomes as any fewer studies may produce unreliable effect size estimates (e.g., Robinson et al., 2022). Multi-level meta-analyses were conducted using the ‘metafor’ package (Harrer et al., 2021). Standardised mean change (SMC) was calculcated using the ‘escalc’ package (Viechtbauer, 2010). With the multi-level I^2^ statistic, 0%, 25%, 50%, and 75% indicate no, low, moderate, and high heterogeneity, respectively (Higgins et al., 2003). Model fit comparing multi-level and conventional meta-analyses were tested using ANOVAs to ensure our data structure reflected true correlations between effects within studies (Harrer et al., 2021). For interpretation purposes, SMC was categorised, where 0.2, 0.5, and 0.8 indicate small, medium, and large effect sizes, respectively (Faraone, 2008). Pooled effects were considered statistically significant if 95% CI did not cross zero; positive effect estimates indicated improvements and negative indicated deteriorations. Where at least 5 comparable studies were available, subgroup analyses on symptom type (e.g., internalising, depressive, anxiety symptoms), diagnostic group (e.g., ADHD, ASD, other SENs), informant (e.g., young person reported, parent reported), and age (e.g., age 10 or above) were conducted. Where appropriate, sensitivity analyses including combined internalising and externalising symptom measures were conducted to assess robustness. We hoped to conduct meta-regressions to assess whether levels of pandemic restrictions influenced changes in mental health but variability in OxCGRT was insufficient.

**Appendix S7** Further information on meta-analyses.

**Pre- versus during pandemic mental health**

*Internalising symptoms*

Conti et al. (2020) used different measures to assess outcomes in 1.5-5- and 6–18-year-olds so they were treated as independent cohorts. Sadeghi et al. (2022) included multiple timepoints across the acute, remission, and resurgence phases, so only one timepoint from each phase was used. Two studies were excluded from meta-analysis due to missing data (De Giacomo et al., 2021; Siracusano et al., 2021).

*Externalising symptoms*

Three studies were excluded from meta-analysis due to missing data (De Giacomo et al., 2021; Siracusano et al., 2021; Suzuki et al., 2021).

**Mental health during the pandemic**

*Internalising symptoms*

Two studies were excluded from meta-analysis due to missing data (Day et al., 2023; Prato et al., 2023) and three were excluded due to timepoints being outside of the two phases (Bouter et al., 2022; Fredrick et al., 2023; Summerton et al., 2023).

**Appendix S8** Further information on narrative synthesis.

**Pre- versus during pandemic mental health**

*Internalising symptoms*

No studies reported change in overall parent (Conti et al., 2020; De Giacomo et al., 2021; de Maat et al., 2023; Lugo-Marin et al., 2021; Siracusano et al., 2021) or young people reported internalising symptoms (Houghton et al., 2022) in children and young people with neurodevelopmental or neuropsychiatric conditions pre-pandemic versus acute (March-May 2020) or remission (July-August 2020) phases.

There was mixed evidence of change in parent and young people reported depressive and anxiety symptoms. For depressive symptoms, most studies found no significant change between pre-pandemic and acute (March-June 2020), remission (July-December 2020), and resurgence (January-March 2021) phases in autistic children and adolescents (Lugo-Marin et al., 2021) or those with depression (Sadeghi et al., 2022), neuropsychiatric (Conti et al., 2020), or neurodevelopmental conditions (Houghton et al., 2022). Bouter et al. (2022) found depressive symptoms increased significantly between pre-pandemic and acute phase (April 2020) in high-risk adolescents overall. Of note, 19.3% of this sample saw an increase in depressive symptoms, while 9.9% saw a decrease and the remaining saw no change.

For anxiety symptoms, most studies found no significant change between pre-pandemic and acute (March-June 2020), remission (July-December 2020), and resurgence (January-March 2021) phases in autistic children and adolescents (Corbett et al., 2021; Lugo-Marin et al., 2021) and those with depression (Sadeghi et al., 2022). One study on children and adolescents with neuropsychiatric conditions found no change in anxiety symptoms in mid-to-late childhood group (aged 6-18 years) but a significant increase in early childhood group (1.5-5 years) between pre-pandemic and acute phase (April-May 2020). Another study on high-risk adolescents found a significant decrease in anxiety symptoms between pre-pandemic and acute phase (April 2020; Bouter et al., 2022). Within the sample, although 8.5% saw a decrease, 8.5% also saw an increase in anxiety symptoms. Change in depressive and anxiety symptoms between pre-pandemic and acute (May-June 2020) or remission (July-August 2020) phases in adolescents with ADHD were not tested statistically in one study (Breaux et al., 2021).

*Externalising symptoms*

Overall, no studies reported change in overall parent (Conti et al., 2020; De Giacomo et al., 2021; de Maat et al., 2023; Lugo-Marin et al., 2021; Siracusano et al., 2021) or young people reported externalising symptoms (Houghton et al., 2022) in autistic children and young people and those with neurodevelopmental or neuropsychiatric conditions pre-pandemic versus acute (March-May 2020) or remission (July-August 2020) phases. Two studies found no change in oppositional defiant problems in high-risk and autistic children and adolescents between pre-pandemic and acute (April-June 2020) or remission (July 2020) phases (Bouter et al., 2022; Lugo-Marin et al., 2021). Of note, Bouter et al., (2022) found that although overall there was no change in oppositional defiant problems, 5.6% of the sample saw an increase and 3.8% a decrease. The single study that assessed irritability found significant decrease in irritability between pre-pandemic and remission phase (October-December 2020) in children and adolescents with neurodevelopmental conditions (Suzuki et al., 2021).

There was mixed evidence for change in parent and young people reported inattention/hyperactivity problems. Two studies found no change between pre-pandemic and acute (April-June 2020) or remission (July 2020) phases in autistic children and adolescents (Lugo-Marin et al., 2021) or those with neuropsychiatric conditions (Conti et al., 2020) and one study found significant decrease in hyperactivity problems between pre-pandemic and remission phase (October-December 2020) in children and adolescents with neurodevelopmental conditions (Suzuki et al., 2021). Change in inattention, hyperactivity/impulsivity, and oppositionality/defiance problems between pre-pandemic and acute (May-June 2020) or remission (July-August 2020) phases in adolescents with ADHD were not tested statistically in one study (Breaux et al., 2021).

*Other symptoms*

One study on children and young people with neuropsychiatric conditions reported a significant increase in parent reported OCD and post-traumatic stress symptoms between pre-pandemic and acute phase (April-May 2020) in mid-to-late childhood group, but not in the early childhood group (Conti et al., 2020). Another reported a reduction in young people reported psychotic symptoms, and suicidality between pre-pandemic and acute (April 2020) in high-risk youth (Bouter et al., 2022). Hall et al. (2022) noted no significant change in clinician-rated tics in children and young people with tic disorders between pre-pandemic and acute/remission phases (March-October 2020). No meta-analysis was conducted due to the low number of studies.

**Mental health during the pandemic**

*Internalising symptoms*

There was mixed evidence for parent and young people reported internalising, depressive, and anxiety symptoms. During the acute phase, one study reported no change between depressive and anxiety symptoms across March-April, April-May, and May 2020 in adolescents with depression (Sadeghi et al., 2022) and two studies did not statistically test change in emotional, depressive, and anxiety symptoms across March-April, April-May, and May-June 2020 in autistic children and adolescents or those with ADHD and other SENDs (Hall et al., 2023; Toseeb & Asbury, 2022). During the remission phase, Day et al. (2023) reported significant reduction in internalising symptoms between June-September and December 2020 in autistic children and adolescents. During the resurgence phase, one study found no significant change in depressive and anxiety symptoms between enrolment (June 2021-June 2022) and 12-month follow-up in children and adolescents with tic disorder (Prato et al., 2023).

Between acute and remission phases, most studies reported no change in internalising, depressive, and anxiety symptoms in autistic children and adolescents (Corbett et al., 2021; Toseeb & Asbury, 2022) or those with neurodevelopmental conditions (Houghton et al., 2022), other SENDs (Toseeb & Asbury, 2022), or depression (Sadeghi et al., 2022). One study reported significant reduction in internalising symptoms between April-May and August-September 2020 in children and adolescents with affective, behavioural, and neurodevelopmental conditions (Raffagnato et al., 2021) and another reported significant decrease in anxiety symptoms between March-April and September-October 2020 in autistic children and other SENDs (Toseeb & Asbury, 2022). Breaux et al. (2021) did not statistically test change in depressive and anxiety symptoms between May-June and July-August 2020 in adolescents with ADHD.

Between acute and resurgence phases, one study found no change in depressive and anxiety symptoms between March-April/April-May/May 2020 and January-March 2021 in adolescents with depression (Sadeghi et al., 2022), while one study found no change in anxiety symptoms but significant increase in depressive symptoms between April 2020 and January 2021 in high-risk adolescents (Bouter et al., 2022). The latter study found variations within their own sample, with 20.5% and 10% showing increase and 7.3% and 4.4% decrease in depressive and anxiety symptoms, respectively. Two studies did not statistically test change in depressive and anxiety symptoms between May-June 2020 and March-June 2021 in children and adolescents with ADHD (Fredrick et al., 2022; Summerton et al., 2023).

Sadeghi et al. (2022) reported no significant change in depressive and anxiety symptoms between remission (June-July/September 2020-January 2021) and resurgence phases (January-March 2021) in adolescents with depression.

*Externalising symptoms*

Most studies reported no change in parent or young people reported externalising, inattention, hyperactivity/impulsivity, and oppositional defiant problems in children and young people with emotional and neurodevelopmental conditions across acute, remission, and resurgence phases. Within the acute phase, Hall et al. (2023) did not statistically test change in conduct and inattention/hyperactivity problems between March-April and April-May 2020 in autistic children and adolescents or those with ADHD. Between acute and remission phases, two studies reported no change in externalising symptoms between March-May and July-September 2020 in children and adolescents with affective, behavioural, and neurodevelopmental conditions (Houghton et al., 2022; Raffagnato et al., 2021). Between acute and resurgence phases, Bouter et al. (2022) reported no change in oppositional defiant problems between April 2020 and January 2021 in high-risk adolescents, of which 2.6% experienced an increase and 4.7% a decrease. Two studies did not statistically test for changes in irritability, inattention, hyperactivity/impulsivity, and oppositional/defiance problems between acute (May-June 2020) and remission phases (July-August 2020) in children and adolescents with ADHD (Breaux et al., 2021; Summerton et al., 2023). No meta-analysis was conducted due to insufficient number of studies to make comparisons across phases.

*Other symptoms*

Findings from parent and young people reported post-traumatic stress symptoms were mostly decreases across acute, remission, and resurgence phases. Lewis et al. (2022) reported significant reduction in post-traumatic stress symptoms between acute (May-June 2020) and remission/resurgence phases (July-August/October-November 2020 and March-May 2021), but no change within/between remission and resurgence phases in adolescents with ADHD or internalising disorders. Another study found post-traumatic stress symptoms significantly reduced between acute (April-May 2020) and remission phases (August-September 2020) in children and adolescents with affective, behavioural, and neurodevelopmental conditions (Raffagnato et al., 2021). With psychotic symptoms and suicidality, Bouter et al. (2022) reported significant decrease in psychotic symptoms but no change in suicidality between acute (April 2020) and resurgence phases (January 2021) in high-risk adolescents. Tic severity and impairment and OCD symptoms were not found to change significantly between enrolment (June 2021-June 2022) and 12-month follow-up in children and adolescents with tic disorder (Prato et al., 2023). Lastly, change in pandemic-related depressive and anxiety symptoms across acute and remission phases were not statistically tested in two studies on autistic children and adolescents or those with ADHD (Dvorsky et al., 2022; Hall et al., 2023). No meta-analysis was conducted due to the low number of studies.

**Explanatory factors**

*Clinical*

Child clinical factors were noted in ten studies. Conti et al. (2020) reported that in 1.5-5-year-old children and adolescents with neuropsychiatric conditions, neurodevelopmental disorder-related problems worsened in children with emotional and behavioural disorders, but anxiety symptoms worsened in those with neurological conditions between pre-pandemic and acute phase (April-May 2020). In 6-18-year-olds, children with emotional and behavioural disorders had worse emotional and behavioural problems than those with neurological and/or neurodevelopmental conditions, and externalising symptoms were worse in children with neurodevelopmental conditions (Conti et al., 2020). One study reported that depressive and anxiety symptoms were worse in autistic children and adolescents compared to those with other SENDs between acute (March-April 2020) and remission phases (September-October 2020; Toseeb & Asbury, 2022). Houghton et al. (2022) found that externalising symptoms decreased between pre-pandemic and remission phase (July/August 2020) in children and adolescents with ADHD, but not those with specific LDs or autistic youth. In children and adolescents with tic disorder, tic severity and impairment between pre-pandemic and March-October 2020 were not influenced by ASD diagnostic status (Hall et al., 2022). Likewise, ADHD or internalising disorder diagnostic status did not have an impact on post-traumatic stress symptom trajectory patterns in adolescents with ADHD or internalising disorders (Lewis et al., 2022).

Three studies looked at pre-pandemic symptom severity, where high symptom severity was mostly associated with better outcomes during the pandemic. Bouter et al. (2022) found that change in depressive and anxiety symptoms, oppositional defiant problems, and psychotic symptoms in high-risk adolescents across pre-pandemic, acute (April 2020), and resurgence phases (January 2021) differed by baseline symptom severity groups, whereby those scoring in the clinical range pre-pandemic had the largest decrease and those in the ‘normal’ range saw small increases. Similarly, Lugo-Marin et al. (2021) reported changes in internalising, anxiety, and depressive symptoms, and oppositional defiant problems between pre-pandemic and acute/remission phases (May-July 2020) were significantly lower in autistic children with increased symptom severity. Another two studies found pre-pandemic symptoms did not influence suicidality (Bouter et al., 2022) and tic severity and impairment (Hall et al., 2022) across pre- and during pandemic phases.

Two studies looked at treatment and one on pre-pandemic emotion regulation. Receiving treatment, such as receiving cognitive behavioural therapy or medication, did not predict changes in depressive and anxiety symptoms in adolescents with depression or ADHD across the pandemic phases (Dvorsky et al., 2022; Sadeghi et al., 2022). Breaux et al. (2021) found that change in inattention and hyperactivity/impulsivity problems across pre-pandemic, acute (May-June 2020), and remission phases (July-August 2020) were significantly increased in adolescents with ADHD with poor pre-pandemic emotion regulation skills.

*Sociodemographic*

Eight studies investigated sociodemographic factors as explanatory factors. The evidence for age as a predictor of outcomes were mixed. Two studies reported that emotional and behavioural problems were worse in older autistic children and adolescents (Toseeb & Asbury, 2022) or those with neuropsychiatric conditions (Conti et al., 2020) across pre-pandemic, acute (March-May 2020), and remission phases (September-October 2020), while another two found outcomes did not differ by age in autistic children and adolescents or those with tic disorder (Hall et al., 2022; Lugo-Marin et al., 2021).

Most evidence suggested that being female predicted worse outcomes. Three studies found that depressive and anxiety symptoms across pre-pandemic, acute, and remission phases were worse in females in autistic adolescents (Toseeb & Asbury, 2022) or those with depression (Sadeghi et al., 2022) or ADHD (Dvorsky et al., 2022). Lewis et al. (2022) reported female adolescents with ADHD or internalising disorder were significantly more likely to have severe fluctuating post-traumatic stress symptoms and significantly less likely to remain in the ‘normal’ range than males between acute (May-June 2020) and resurgence phases (March-May 2021). However, sex had no impact on other post-traumatic stress symptom trajectories. Two studies reported no impact of sex on depressive and anxiety symptoms in adolescents with ADHD (Fredrick et al., 2022) or tic severity and impairment in adolescents with tic disorder (Hall et al., 2022), respectively.

There was mixed evidence for the influence of financial hardship, but ethnicity was not found to predict outcomes. Conti et al. (2020) noted between pre-pandemic and acute phase (April-May 2020), internalising, post-traumatic stress, and obsessive-compulsive symptoms were worse in children with neuropsychiatric disorders with increased financial hardship. However, Toseeb & Asbury (2022) found depressive and anxiety symptoms were not impacted by income or ethnicity in autistic children and adolescents and other SENDs. Dvorsky et al. (2022) reported that race or ethnicity did not predict pandemic-related depressive and anxiety symptoms in adolescents with ADHD across acute (May-June 2020) and remission phases (July-August/October-November 2020).

*Pandemic-related*

Pandemic-related factors were highlighted in five studies. There was some evidence for COVID-19 stress and its impact; Summerton et al. (2023) reported COVID-19 stress in acute phase (May 2020) was significantly associated with increased hyperactivity/impulsivity problems in resurgence phase (May-June 2021), but not with depressive symptoms, anxiety symptoms, irritability, inattention, or oppositional defiant problems in children and adolescents with ADHD. Lewis et al. (2022) found adolescents with ADHD or internalising disorders who reported higher COVID-19 impact were significantly more likely to have moderate or clinical post-traumatic stress symptoms across acute (May-June 2020) and resurgence phases (March-May 2021).

Education and engagement with coping mechanisms were reported to be associated with outcomes in two studies. Toseeb & Asbury (2022) noted that depressive symptoms, but not anxiety, were worse in autistic children and adolescents and other SENDs who were in mainstream school during the pandemic compared to those who had alternate provision. However, receiving an education, health and care plan (EHCP) had no impact on depressive and anxiety symptoms. Engagement in daily routines, activities, and structure in acute phase (May-June 2020) significantly predicted pandemic-related depressive and anxiety symptoms in remission phases (July-August/October-November 2020) in adolescents with ADHD (Dvorsky et al., 2022).

There was no evidence for parental mental health and social connectedness as explanatory factors; de Maat et al. (2023) reported they did not significantly predict emotional and behavioural problems in autistic children and adolescents between pre-pandemic and resurgence phase (January-May 2021).

**Appendix S9** Egger’s regression tests of funnel plot asymmetry.

**Pre- vs during COVID**

*Internalising*

Test result: t = 0.67, df = 23, p-value = 0.5091

Bias estimate: 0.8828 (SE = 1.3161)

The intercept of the regression model is 0.8828. This is non-significantly larger than zero (t = 0.67, p = .5091) which indicates that the data in the funnel plot is not asymmetrical. Thus, there is no strong evidence of publication bias.

*Externalising*

Test result: t = 1.24, df = 11, p-value = 0.2422

Bias estimate: 2.7425 (SE = 2.2187)

The intercept of the regression model is 2.7425. This is non-significantly larger than zero (t = 1.24, p = .2422) which indicates that the data in the funnel plot is not asymmetrical. Thus, there is no strong evidence of publication bias.

**During COVID**

*Internalising*

Test result: t = 0.18, df = 11, p-value = 0.8577

Bias estimate: 0.1405 (SE = 0.7654)

The intercept of the regression model is 0.1405. This is non-significantly larger than zero (t = 0.18, p = .8577) which indicates that the data in the funnel plot is not asymmetrical. Thus, there is no strong evidence of publication bias.

**
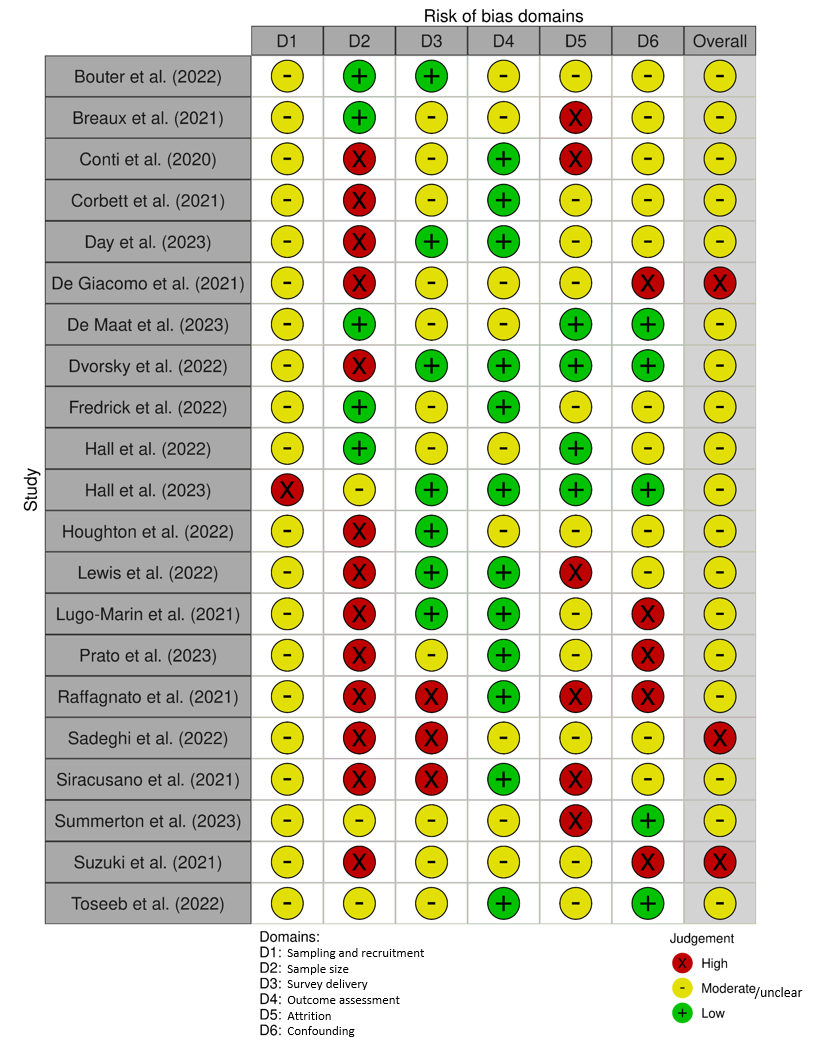
**

**Figure S2.** Traffic light plot of risk of bias of included studies.


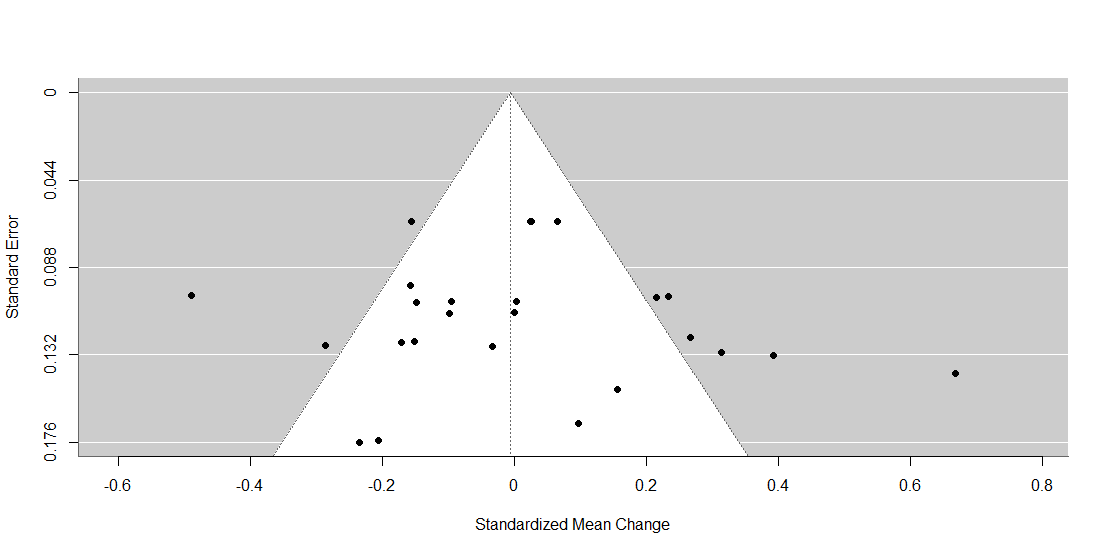


**Figure S3.** Funnel plot of studies assessing internalising symptoms pre- versus during pandemic.


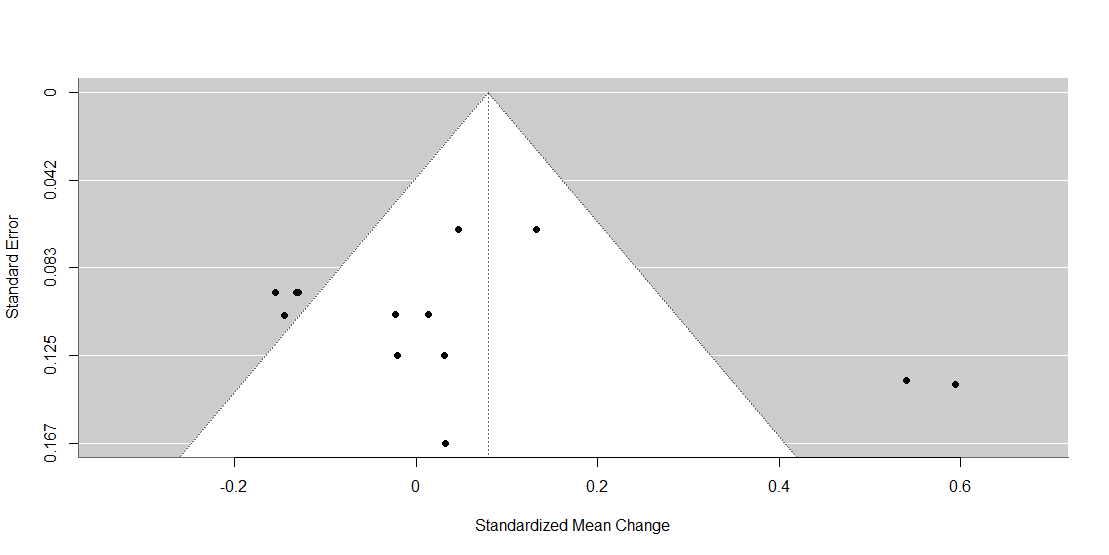


**Figure S4.** Funnel plot of studies assessing externalising symptoms pre- versus during pandemic.


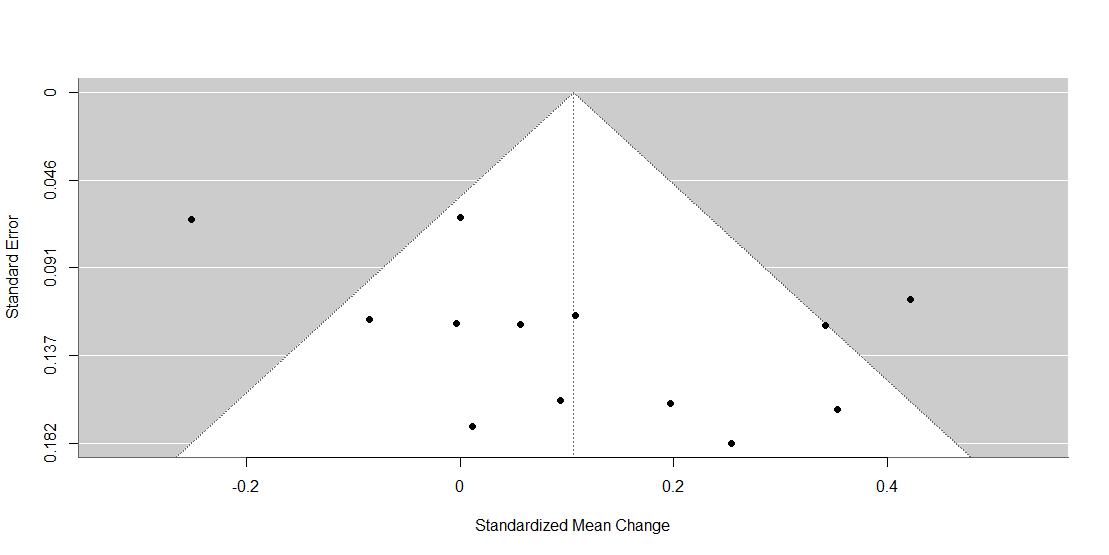


**Figure S5.** Funnel plot of studies assessing internalising symptoms during pandemic.

**Table S1** Multi-level I^2^ distribution for internalising symptoms between pre- and during pandemic.

|  | % of total variance | I^2^ |
| --- | --- | --- |
| Level 1 | 18.95149 |  |
| Level 2 | 19.11631 | 19.12 |
| Level 3 | 61.93220 | 61.93 |
| Total I^2^ | 81.05 |  |

**Table S2** Meta-analysis model comparison for internalising symptoms between pre- and during pandemic.

|  | df | AIC | BIC | LRT | p |
| --- | --- | --- | --- | --- | --- |
| Full | 3 | -3.2395 | 0.2946 |  |  |
| Reduced | 2 | 4.1732 | 6.5294 | 9.4128 | 0.002 |


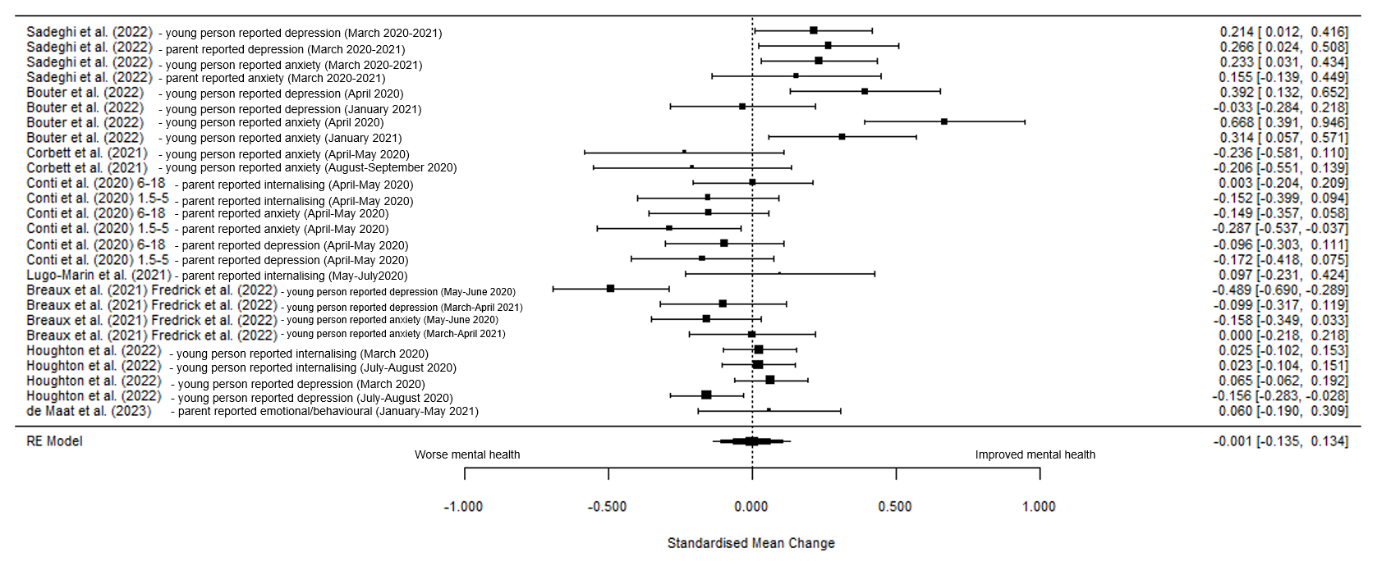


**Figure S6.** Forest plot of sensitivity analysis of the inclusion of a study with a combined emotional and behavioural problem score for internalising symptoms pre- versus during pandemic.


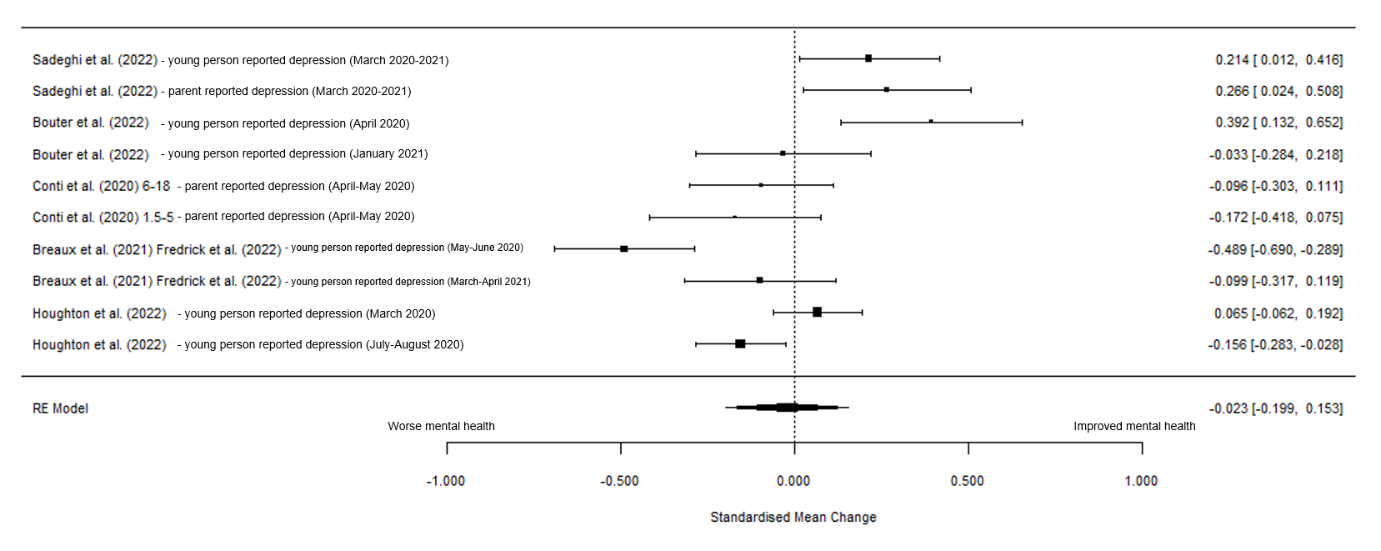


**Figure S7.** Forest plot of subgroup analysis by depressive symptoms for internalising symptoms pre-versus during pandemic.


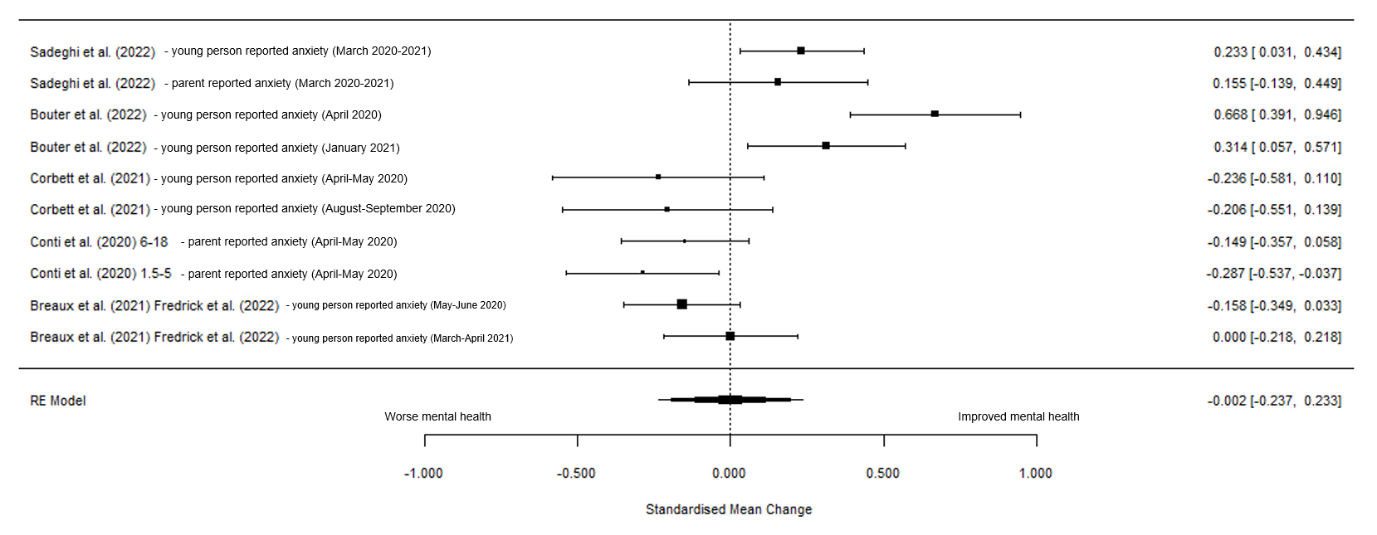


**Figure S8.** Forest plot of subgroup analysis by anxiety symptoms for internalising symptoms pre- versus during pandemic.

**
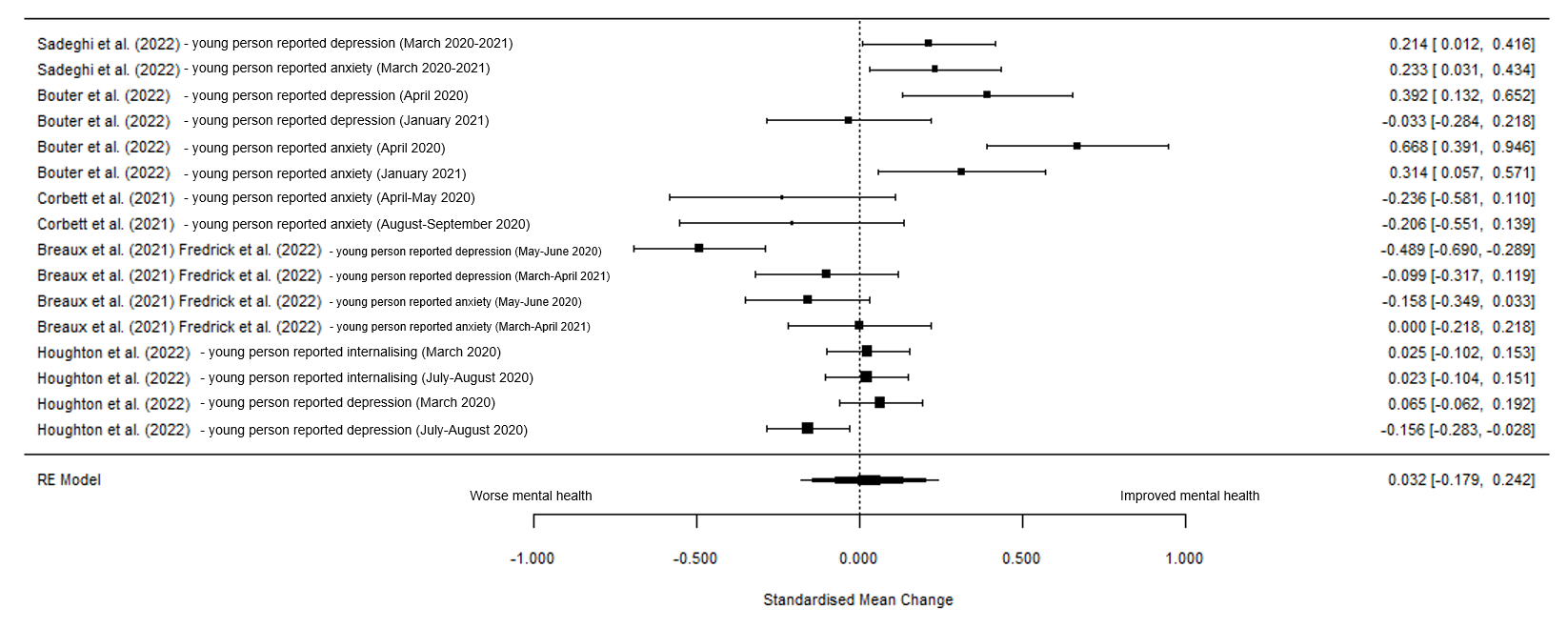
**

**Figure S9.** Forest plot of subgroup analysis by young people reported data for internalising symptoms pre- versus during pandemic.


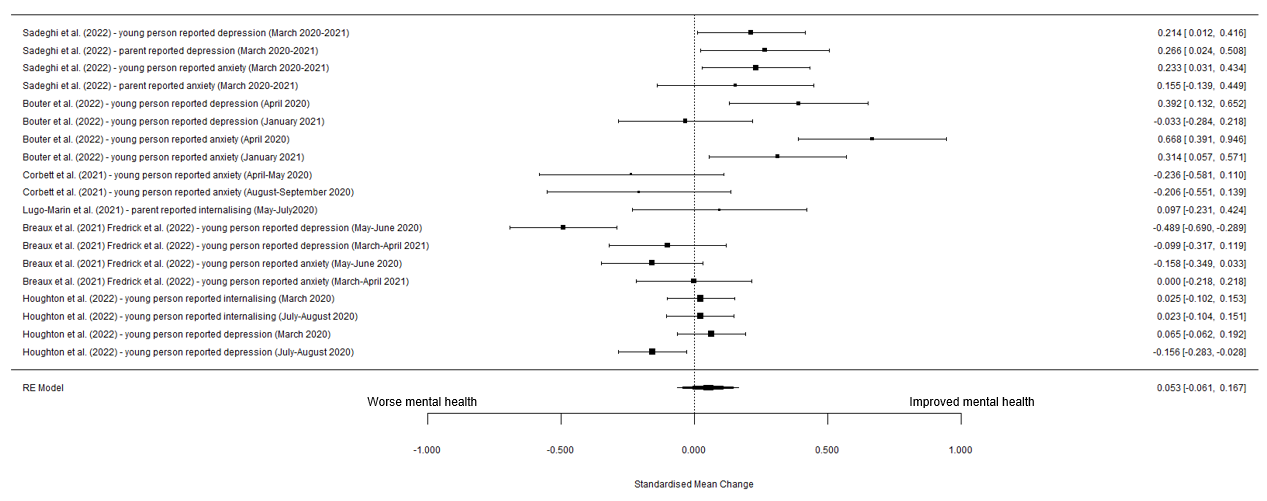


**Figure S10.** Forest plot of subgroup analysis by age 10 years or over for internalising symptoms pre- versus during pandemic.


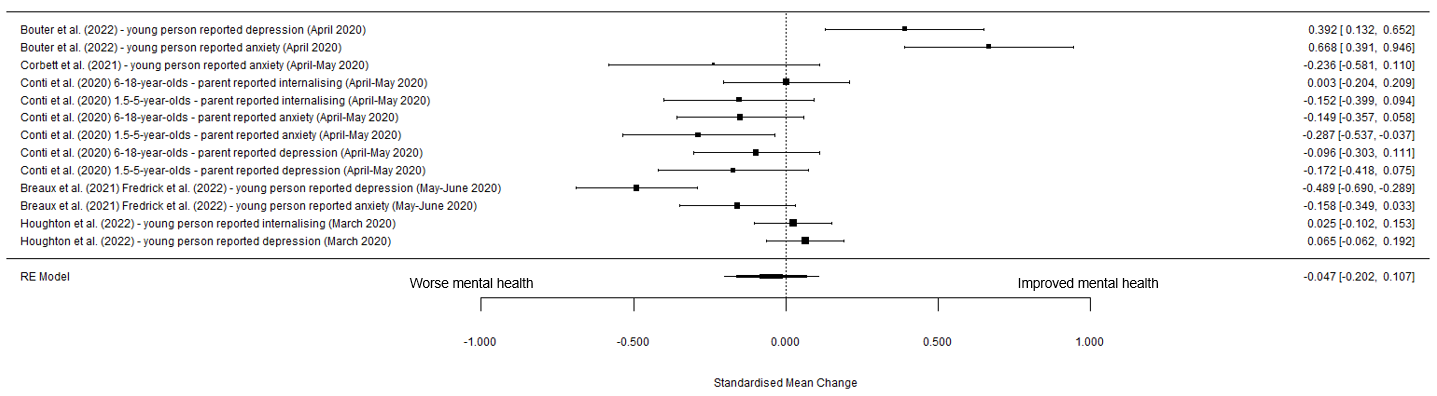


**Figure S11.** Forest plot of subgroup analysis by acute phase for internalising symptoms during pandemic.

**Table S3** Multi-level I^2^ distribution for externalising symptoms between pre- and during pandemic.

|  | % of total variance | I^2^ |
| --- | --- | --- |
| Level 1 | 1.557365e+01 |  |
| Level 2 | 4.403013e-09 | 0 |
| Level 3 | 8.442635e+01 | 84.43 |
| Total I^2^ | 84.43 |  |

**Table S4** Meta-analysis model comparison for externalising symptoms between pre- and during pandemic.

|  | df | AIC | BIC | LRT | p |
| --- | --- | --- | --- | --- | --- |
| Full | 3 | -6.9827 | -5.5280 |  |  |
| Reduced | 2 | 3.0717 | 4.0415 | 12.0544 | 0.0005 |


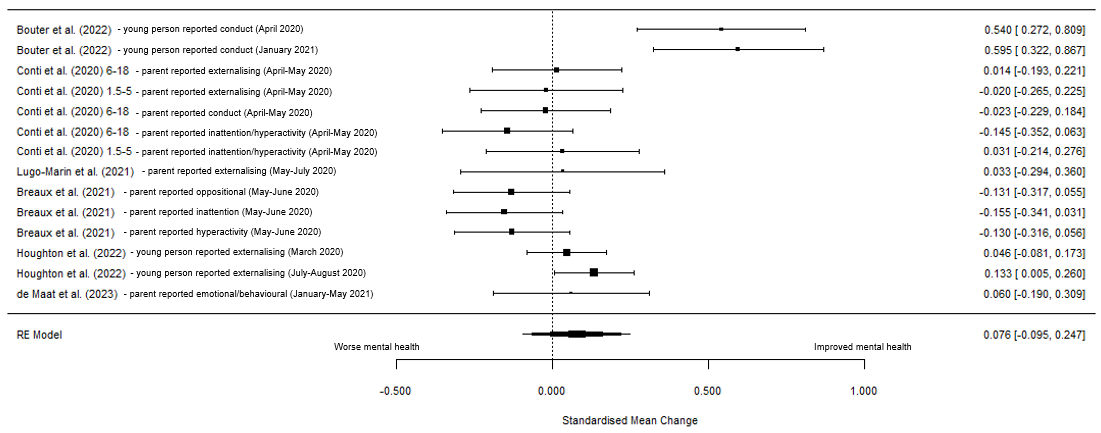


**Figure S12.** Forest plot of sensitivity analysis of the inclusion of a study with a combined emotional and behavioural problem score for externalising symptoms pre- versus during pandemic.

**Table S5** Multi-level I^2^ distribution for internalising symptoms between during pandemic timepoints.

|  | % of total variance | I^2^ |
| --- | --- | --- |
| Level 1 | 25.50260 |  |
| Level 2 | 14.46222 | 14.46 |
| Level 3 | 60.03518 | 60.04 |
| Total I^2^ | 74.50 |  |

**Table S6** Meta-analysis model comparison for internalising symptoms between during pandemic timepoints.

|  | df | AIC | BIC | LRT | p |
| --- | --- | --- | --- | --- | --- |
| Full | 3 | -1.2069 | 0.2478 |  |  |
| Reduced | 2 | -0.0376 | 0.9323 | 3.1694 | 0.0750 |


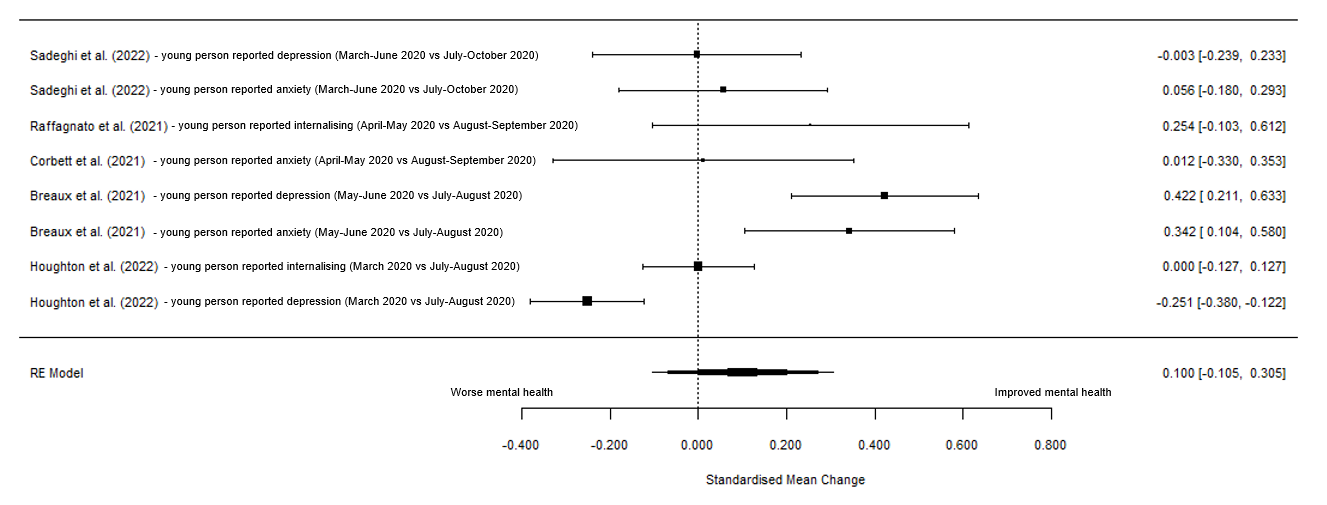


**Figure S13.** Forest plot of subgroup analysis by young people reported data for internalising symptoms between acute and remission phases.


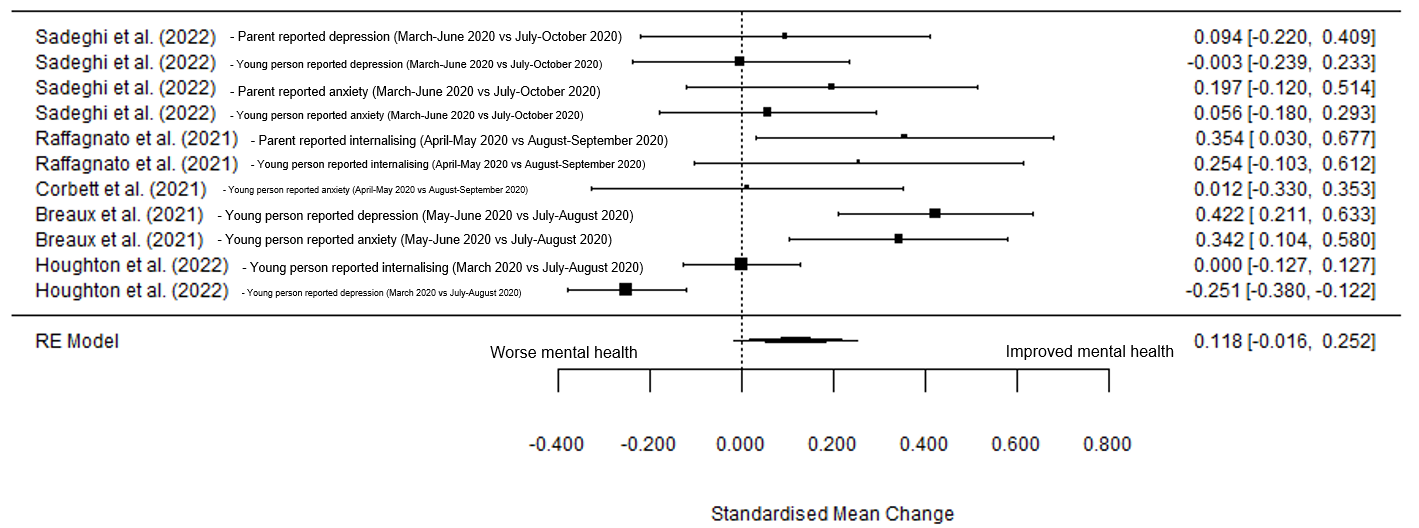


**Figure S14.** Forest plot of subgroup analysis by yage 10 years or above for internalising symptoms between acute and remission phases.

## References:

Achenbach, T. M. (2001). Manual for ASEBA school-age forms & profiles. *University of Vermont, Research Center for Children, Youth & Families.*

Bouter, D. C., Zarchev, M., de Neve-Enthoven, N. G. M., Ravensbergen, S. J., Kamperman, A. M., Hoogendijk, W. J. G., & Grootendorst-van Mil, N. H. (2023). A longitudinal study of mental health in at-risk adolescents before and during the COVID-19 pandemic. *European Child & Adolescent Psychiatry, 32*(6), 1109-1117.

Breaux, R., Dvorsky, M. R., Marsh, N. P., Green, C. D., Cash, A. R., Shroff, D. M., ... & Becker, S. P. (2021). Prospective impact of COVID‐19 on mental health functioning in adolescents with and without ADHD: Protective role of emotion regulation abilities. *Journal of Child Psychology and Psychiatry, 62*(9), 1132-1139.

Chorpita, B. F., Moffitt, C. E., & Gray, J. (2005). Psychometric properties of the Revised Child Anxiety and Depression Scale in a clinical sample. *Behaviour Research and Therapy, 43*(3), 309-322.

Conti, E., Sgandurra, G., De Nicola, G., Biagioni, T., Boldrini, S., Bonaventura, E., ... & Battini, R. (2020). Behavioural and emotional changes during covid-19 lockdown in an italian paediatric population with neurologic and psychiatric disorders. *Brain Sciences, 10*(12), 918.

Corbett, B. A., Muscatello, R. A., Klemencic, M. E., & Schwartzman, J. M. (2021). The impact of COVID‐19 on stress, anxiety, and coping in youth with and without autism and their parents. *Autism Research, 14*(7), 1496-1511.

Day, T. C., Gerber, A., McNair, M. L., Reicher, D., & Lerner, M. D. (2023). Trajectories of internalizing symptoms among autistic and nonautistic youth during the COVID‐19 pandemic. *Autism Research.*

De Giacomo, A., Pedaci, C., Palmieri, R., Simone, M., Costabile, A., & Craig, F. (2021). Psychological impact of the SARS-CoV-2 pandemic in children with neurodevelopmental disorders and their families: Evaluation before and during COVID-19 outbreak among an Italian sample. *Rivista Di Psichiatria, 56*(4), 205-210.

de Maat, D. A., Van der Hallen, R., de Nijs, P. F., Visser, K., Bastiaansen, D., Truijens, F. L., ... & Dekker, L. P. (2023). Children with Autism Spectrum Disorder in Times of COVID-19: Examining Emotional and Behavioral Problems, Parental Well-Being, and Resilience. *Journal of Autism and Developmental Disorders*, 1-12.

Dvorsky, M. R., Breaux, R., Cusick, C. N., Fredrick, J. W., Green, C., Steinberg, A., ... & Becker, S. P. (2022). Coping with COVID-19: longitudinal impact of the pandemic on adjustment and links with coping for adolescents with and without ADHD. *Research on Child and Adolescent Psychopathology, 50*(5), 605-619.

Faraone, S. V. (2008). Interpreting estimates of treatment effects: implications for managed care. *Pharmacy and Therapeutics, 33*(12), 700.

Fredrick, J. W., Nagle, K., Langberg, J. M., Dvorsky, M. R., Breaux, R., & Becker, S. P. (2022). Rumination as a mechanism of the longitudinal association between COVID-19-related stress and internalizing symptoms in adolescents. *Child Psychiatry & Human Development*, 1-10.

Goodman, A., & Goodman, R. (2009). Strengths and difficulties questionnaire as a dimensional measure of child mental health. *Journal of the American Academy of Child & Adolescent Psychiatry, 48*(4), 400-403.

Hale, T., Angrist, N., Goldszmidt, R., Kira, B., Petherick, A., Phillips, T., ... & Tatlow, H. (2021). A global panel database of pandemic policies (Oxford COVID-19 Government Response Tracker). *Nature Human Behaviour, 5*(4), 529-538.

Hall, C. L., Partlett, C., Valentine, A. Z., Pearcey, S., & Sayal, K. (2023). Understanding the impact of home confinement on children and young people with ADHD and ASD during the COVID-19 pandemic. *Child Psychiatry & Human Development*, 1-15.

Harrer, M., Cuijpers, P., Furukawa, T. A., & Ebert, D. D. (2021). Doing Meta-Analysis with R: A Hands-On Guide. Boca Raton, FL and London: Chapmann & Hall/CRC Press. ISBN 978-0-367-61007-4.

Higgins, J. P., Thompson, S. G., Deeks, J. J., & Altman, D. G. (2003). Measuring inconsistency in meta-analyses. *BMJ, 327*(7414), 557-560.

Houghton, S., Kyron, M., Lawrence, D., Hunter, S. C., Hattie, J., Carroll, A., ... & Chen, W. (2022). Longitudinal trajectories of mental health and loneliness for Australian adolescents with‐or‐without neurodevelopmental disorders: the impact of COVID‐19 school lockdowns. *Journal of Child Psychology and Psychiatry, 63*(11), 1332-1343.

Leckman, J. F., Riddle, M. A., Hardin, M. T., Ort, S. I., Swartz, K. L., Stevenson, J. O. H. N., & Cohen, D. J. (1989). The Yale Global Tic Severity Scale: initial testing of a clinician-rated scale of tic severity. *Journal of the American Academy of Child & Adolescent Psychiatry, 28*(4), 566-573.

Lewis, J., Jayakumar, S., Breaux, R., Dvorsky, M. R., Langberg, J. M., & Becker, S. P. (2023). Prospective examination of psychological trauma among adolescents during the COVID-19 pandemic. *Psychological Trauma: Theory, Research, Practice, and Policy*, 15(3), 404.

Lugo-Marín, J., Gisbert-Gustemps, L., Setien-Ramos, I., Español-Martín, G., Ibañez-Jimenez, P., Forner-Puntonet, M., ... & Ramos-Quiroga, J. A. (2021). COVID-19 pandemic effects in people with Autism Spectrum Disorder and their caregivers: Evaluation of social distancing and lockdown impact on mental health and general status. *Research in Autism Spectrum Disorders, 83*, 101757.

Nikolaidis, A., Paksarian, D., Alexander, L., Derosa, J., Dunn, J., Nielson, D. M., ... & Merikangas, K. R. (2021). The Coronavirus Health and Impact Survey (CRISIS) reveals reproducible correlates of pandemic-related mood states across the Atlantic. *Scientific Reports, 11*(1), 8139.

Prato, A., Saia, F., Milana, M. C., Scerbo, M., Barone, R., & Rizzo, R. (2023). Functional tic-like behaviours during the COVID-19 pandemic: Follow-up over 12 months. *Frontiers in Pediatrics, 10*, 1003825.

Raffagnato, A., Iannattone, S., Tascini, B., Venchiarutti, M., Broggio, A., Zanato, S., ... & Gatta, M. (2021). The COVID-19 pandemic: a longitudinal study on the emotional-behavioral sequelae for children and adolescents with neuropsychiatric disorders and their families. *International Journal of Environmental Research and Public Health, 18*(18), 9880.

Robinson, E., Sutin, A. R., Daly, M., & Jones, A. (2022). A systematic review and meta-analysis of longitudinal cohort studies comparing mental health before versus during the COVID-19 pandemic in 2020. *Journal of Affective Disorders, 296*, 567-576.

Sadeghi, N., Fors, P. Q., Eisner, L., Taigman, J., Qi, K., Gorham, L. S., ... & Nielson, D. M. (2022). Mood and behaviors of adolescents with depression in a longitudinal study before and during the COVID-19 pandemic. *Journal of the American Academy of Child & Adolescent Psychiatry, 61*(11), 1341-1350.

Siracusano, M., Segatori, E., Riccioni, A., Emberti Gialloreti, L., Curatolo, P., & Mazzone, L. (2021). The impact of COVID-19 on the adaptive functioning, behavioral problems, and repetitive behaviors of Italian children with autism spectrum disorder: An observational study. *Children, 8*(2), 96.

Summerton, A., Bellows, S. T., Westrupp, E. M., Stokes, M. A., Coghill, D., Bellgrove, M. A., ... & Sciberras, E. (2023). Longitudinal Associations Between COVID-19 Stress and the Mental Health of Children With ADHD. *Journal of Attention Disorders*, 10870547231168334.

Suzuki, K., & Hiratani, M. (2021). Impact of the COVID-19 pandemic on children with neurodevelopmental disorders when school closures were lifted. *Frontiers in Pediatrics, 9*, 789045.

Toseeb, U., & Asbury, K. (2023). A longitudinal study of the mental health of autistic children and adolescents and their parents during COVID-19: Part 1, quantitative findings. *Autism, 27*(1), 105-116.

Viechtbauer, W. (2010). Conducting meta-analyses in R with the metafor package. Journal of Statistical Software, 36(3), 1-48.
